# Bistability in Glycolysis Pathway as a Physiological Switch in Energy Metabolism

**DOI:** 10.1371/journal.pone.0098756

**Published:** 2014-06-09

**Authors:** Bhanu Chandra Mulukutla, Andrew Yongky, Prodromos Daoutidis, Wei-Shou Hu

**Affiliations:** Department of Chemical Engineering and Materials Science, University of Minnesota, Minneapolis, Minnesota, United States of America; Mayo Clinic, United States of America

## Abstract

The flux of glycolysis is tightly controlled by feed-back and feed-forward allosteric regulations to maintain the body's glucose homeostasis and to respond to cell's growth and energetic needs. Using a mathematical model based on reported mechanisms for the allosteric regulations of the enzymes, we demonstrate that glycolysis exhibits multiple steady state behavior segregating glucose metabolism into high flux and low flux states. Two regulatory loops centering on phosphofructokinase and on pyruvate kinase each gives rise to the bistable behavior, and together impose more complex flux control. Steady state multiplicity endows glycolysis with a robust switch to transit between the two flux states. Under physiological glucose concentrations the glycolysis flux does not move between the states easily without an external stimulus such as hormonal, signaling or oncogenic cues. Distinct combination of isozymes in glycolysis gives different cell types the versatility in their response to different biosynthetic and energetic needs. Insights from the switch behavior of glycolysis may reveal new means of metabolic intervention in the treatment of cancer and other metabolic disorders through suppression of glycolysis.

## Introduction

Glycolysis is the conduit of glucose metabolism for generating energy and providing biosynthetic precursors for cellular materials. The flux of glycolysis in cancer cells is high as compared to normal adult tissues and a vast amount of the glucose consumed is diverted towards lactate production; a phenomenon known as the Warburg effect [1]. This behavior is also typical of highly proliferative tissues such as fetal tissues and stem cells [2], [3]). In contrast, quiescent cells transport glucose at low rates and catabolize most glucose to carbon dioxide [4], [5]. The Warburg effect was previously attributed to defective oxidative phosphorylation [1], [6] until it was realized that the pathway was not impaired in most tumor cells (see review [7]).

The differences in glycolysis activity observed across various cell types is accomplished through different levels of regulation [8]. At one such level is the allosteric feed-back and feed-forward regulations exerted by the intermediate metabolites on its enzymes. Pivotal roles are played by three enzymes, (phosphofructokinase (PFK), pyruvate kinase (PK) and phosphofructokinase/fructose-2,6-bisphosphatase (PFKFB)) through their inhibition or activation by three reaction intermediates (fructose-1,6-bisphosphate (F16BP), fructose-2,6-bisphosphate (F26BP), and phosphoenolpyruvate (PEP)) in glycolysis. These enzymes have multiple isoforms (PFKL/M/P, PKM1/M2/L/R and PFKFB1-4) which are subjected to contrasting allosteric regulations [9]–[11]. Each isoform, therefore, affects the glycolytic activity in a distinct manner.

All three isoforms of PFK are activated by F6P and F26BP [12], but only PFKM and PFKL are activated by F16BP [13]–[15]. PFKFB is a bifunctional enzyme whose kinase and bisphosphatase domains catalyze the formation and hydrolysis reaction of F26BP, respectively [9], [16]. Isozymes of PFKFB differ in their kinase and phosphatase activities as well as in their sensitivity to feedback inhibition by phosphoenolpyruvate (PEP) [17]–[19]. Thus, each isozyme of PFKFB has a profoundly distinct capacity in modulating PFK activity. Pyruvate kinase (PK) in mammalian systems is encoded by two genes that can produce two isoforms each. Except for the PKM1 isoform, the other three isoforms of PK, PKM2, PKL and PKR, are activated by F16BP to varying extents [11]. The M2 isoform of PK, in addition to activation by F16BP, is also under the control of a host of allosteric modulators including serine, succinylaminoimidazolecarboxamide ribose-5-phosphate (SAICAR) and phenylalanine among others [20]–[22]. The sensitivity of the M2 isoform to such wide ranging modulators allows it to act, in-part, as the cell's nutrient sensing machinery [20]. Different tissues and cell types express different isoform combinations of these enzymes, thus giving rise to a suitable glycolytic flux behavior that caters to the biosynthetic and energetic needs of the cell type in question.

The expression of isoforms of glycolytic enzymes and their regulation is tightly linked to control of cell growth [23]. The make-up of the glycolytic isoforms in quiescent tissues strictly restrains its flux, thus restricting the provision of the carbon for growth and proliferation. There is increasing evidence that loss of the growth control as in the case of tumor formation caused by mutations in proto-oncogenes and tumor suppressors, is accompanied by alteration in the expression of specific glycolytic isozymes leading to metabolic reprogramming [24], [25]. For example, HK2 is only expressed in limited number of adult tissues but is expressed at high levels in cancer cells. HK2 binds to outer mitochondrial membrane and inhibits the release of cytochrome C to suppress apoptosis and promotes cell survival in cancer cells [26], [27]. Analogously, the embryonic isoform of pyruvate kinase, PKM2, is found to be expressed in few adult tissues, but is known to be highly expressed across wide range of tumor cells. Interestingly, knockdown of PKM2 in cancer cells, such as the human lung cancer cell line H1299, and replacing it with PKM1 was demonstrated to result in a metabolic phenotype change involving decreased glucose uptake and increased oxidative phosphorylation [28]. Further, reprogramming of somatic cells to induced pluripotent stem cells (iPSCs) has also been shown to incur metabolic reprogramming; the change from a low glycolytic flux state of somatic cells to a high flux state of rapidly dividing iPSC cells is accompanied by a switch in the isozyme expression of HK and PFK enzymes [4].

An additional layer of flux regulation of glycolysis is exerted by signaling pathways. Through signaling pathways, contrasting glycolysis flux behavior is accomplished without changing the isoforms [7], [23]; instead the action of signaling pathways alters the kinetic behavior of the target enzyme. Tyrosine kinase signaling has been shown to change the kinetic behavior of PKM2 isoform through modulation of its allosteric regulations [28], [29]. Similarly, signaling events triggered by glucagon in hepatocytes alter the kinetics of the liver isozyme of PK [30]. Protein kinases A/B/C (PKA, PKB and PKC) have been shown to affect the kinetics of PFKFB isoforms [16].

The composition of isozymes in glycolysis, through multiple layers of regulation, is pivotal to the flux control and plays a key role in growth control and physiological balance. Over the last four decades, the kinetic behavior of isoforms of individual glycolytic enzymes has been examined in detail. However, a holistic understanding of the effect of different combinations of such isoforms on the flux behavior of the complete glycolysis pathway is yet to be attained. We have taken a systems biology approach to study the flux states of glycolysis pathway. Using a mathematical model that employs mechanistic rate equations for enzyme kinetics, we demonstrate that glycolysis exhibits a classical multiple steady state behavior in terms of its flux with respect to the glucose concentration. The multiplicity of steady states segregate cell metabolism into distinct states: high glycolytic flux states and low glycolytic flux states. Such bistable behavior is an output of complex allosteric regulations which in turn depend on the type of glycolytic isozymes expressed. We show that the presence of the muscle or the liver isozyme of PFK or/and the L, R or M2 isoform of PK is necessary for multistability in glycolytic flux. We substantiated the modeling insights with gene expression data from various tissues as well as experimental data from HeLa cells. Further, we discuss the factors that affect the bistable nature of the glycolysis such as the level and the K/P ratio of enzyme PFKFB.

Similar kinds of bistable behavior have been shown to act like a robust switch in many regulatory circuits including oocyte cell maturation [31], transition from quiescent to proliferative modes in mammalian cells [32], transition between multiple phospho-form stable states in multisite phosphorylation systems [33], among many others. The dissection of glycolytic flux as a bistable switch will provide new insights on the regulation of cell metabolism and possibly allow for a new perspective in identifying ways to modulate metabolic activities for therapeutic purposes.

## Results

In the following sections we will first present the steady state behavior of the F6P-node, and then discuss the effect of the regulatory behavior of F6P-node on the glycolysis flux. This is followed by description and analysis of the effect of a second regulatory loop acting on the glycolysis pathway. Lastly, the combined effect of the two loops will be discussed.

### Bistability in the F6P-node

**Figure 1:**
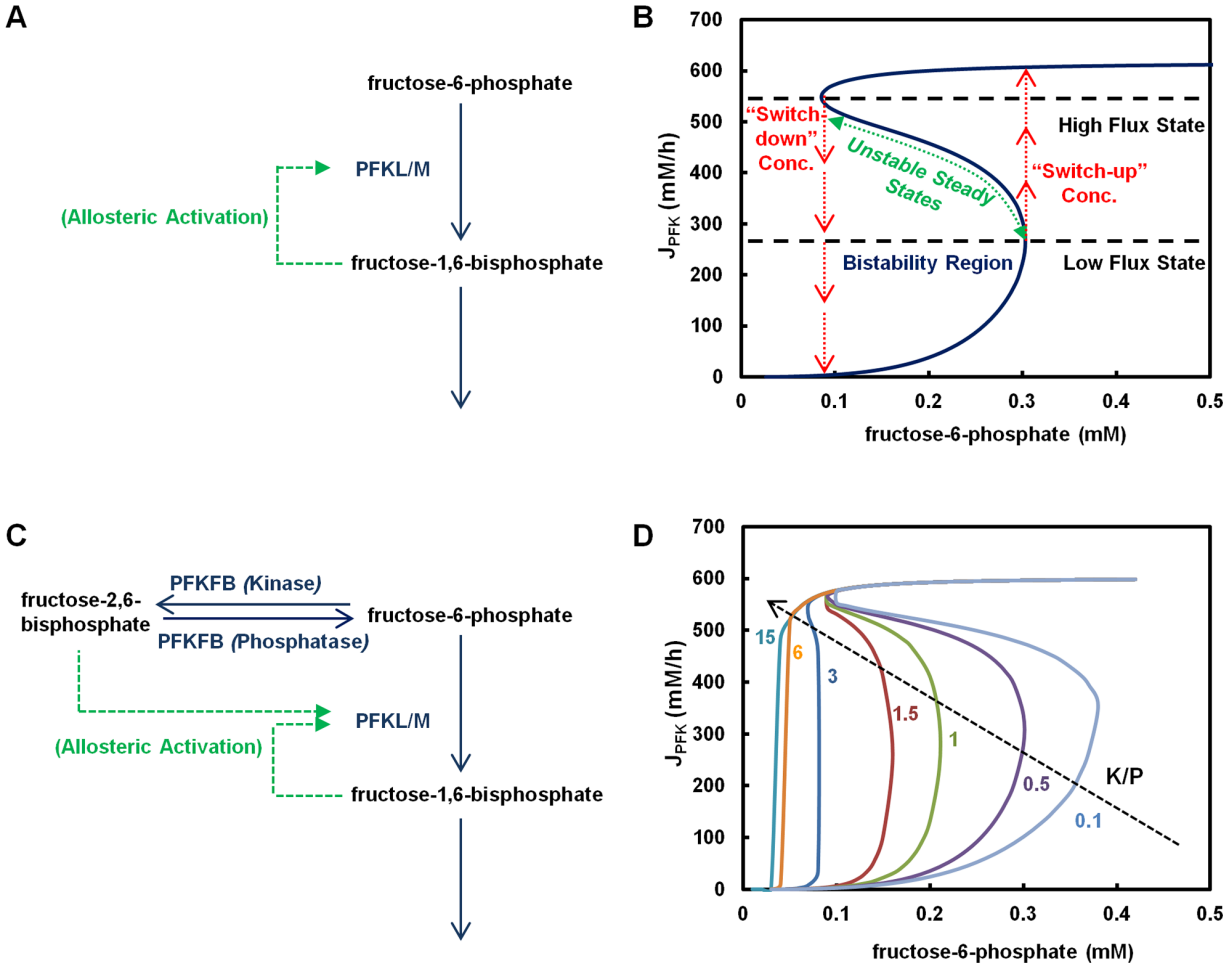
Multiplicity of steady states in kinetics of fructose-6-phosphate node (F6P-node). (**A**) Feedback activation of phosphofructokinase (PFK) by fructose-1,6-bisphosphate (F16BP) (**B**) Bistability in the kinetics of PFK due to feedback regulation by F16BP. The simulation was performed using only two enzymes PFK and aldolase (ALDO). F6P was varied and the steady state PFK flux was solved algebraically. Activation of PFK by F16BP was set at ***K_PFK,fbp_***  = 0.65 mM. (**C**) Allosteric regulations in the F6P-node. (**D**) Modulation of bistability span by K/P ratio of PFKFB. The bifunctional enzyme PFKFB was integrated with PFK and ALDO to construct a three enzyme system. Simulations were performed at varying K/P value of PFKFB while keeping all other conditions the same as in (**B**).

Phosphofructokinase (PFK) is a pivotal enzyme in glycolysis and exists in three distinct isoforms. The muscle (PFKM) and the liver (PFKL) isozymes are under allosteric feedback activation by F16BP to a varying extent [13], [14], [34], whereas the platelet isozyme (PFKP) is not [35]. It is well known that feedback activation can give rise to ultrasensitivity and even bistability. To assess the effect of the allosteric regulation of F16BP on the PFK flux, we constructed a mechanistic model around the F6P-node encompassing the reactions catalyzed by the enzymes PFK and aldolase (ALDO) (Figure 1A). The values of kinetic parameters for each enzyme were obtained from various literatures in which the values have been experimentally determined [12], [14], [36]. The simulated steady state behavior is thus only affected by the relative abundance levels of the two enzymes involved. The relative enzyme levels were obtained from their corresponding transcript levels in cultured cells and assuming that the protein level is proportional to the transcript [37]. Furthermore, the concentrations of DHAP and GAP were held at constant levels of 0.04 mM and 0.02 mM, respectively.

In the absence of feedback activation by F16BP as is the case for PFKP, the steady state flux resembles a Michaelis-Menten type of kinetics (Figure S1). In contrast, the activation by F16BP on PFK as in the case of the isozyme PFKL (or PFKM) ([12], [14]), causes the steady state flux of PFK at different F6P concentrations (Figure 1B) to bear the hallmark of bistability. In the region bound by F6P concentrations from 0.09 mM to 0.3 mM, three types of steady state can be seen, two of which are stable (low flux states and high flux states) and the ones in the middle are unstable. The physiological concentration of F6P in rat liver tissue has been reported to be ∼0.1 mM [38]. Outside the region, only one steady state for a given F6P concentration is observed; below 0.09 mM the PFK steady state flux exists only in the low state, whereas above 0.3 mM it exists only at the high state. Eigenvalue analysis confirmed the stability of each steady state. It should be noted that the above specified concentration ranges for bistable behavior are subject to the model parameters (kinetic constants and enzyme levels) of the F6P-node.

In the bistable region, the flux can be either at a low or a high state depending on the previous state of the system. The in-between states are unstable in nature; these states are never realized experimentally. When the concentration of F6P is varied slowly, the PFK flux changes along the stable steady state lines. Starting at a low flux state, as the F6P concentration increases, the flux increases along the low flux steady state line until the F6P concentration reaches 0.3 mM (“switch-up” concentration, Figure 1B), and then the system undergoes a sharp transition to a high flux state. Further increase in F6P moves the system farther up along the high flux steady state line. Conversely, if the system is initially at a high flux state, as the F6P concentration decreases, the flux remains at the high state until the F6P concentration decreases to 0.09 mM (“switch-down” concentration, Figure 1B) where it rapidly descends to the low state. Once the system is switched from the low flux state to the high flux state, or vice versa, it does not switch back to the original state by small fluctuations of F6P concentration at the switch-concentration. The system is thus marked by well separated high flux and low flux states, and very distinctive “switch-up” and “switch-down” F6P concentrations.

All three isozymes of PFK are activated by F26BP. The bi-functional enzyme PFKFB catalyzes both the formation and degradation of F26BP (Figure 1C). The steady state concentration of F26BP is thus not affected by the expression level of PFKFB but by the balance between the relative activities of the kinase (K) and the bisphosphatase (P) domains of PFKFB. Thus, the flux through PFK is indirectly influenced by the K to P activity ratio of PFKFB, also termed as the K/P ratio.

We examined the regulation of the PFK flux by incorporating PFKFB into the model of the F6P-node. The experimentally determined values of kinetic parameters for PFKFB were also obtained from literature [39], [40]. The K/P ratio of PFKFB alters the steady state behavior of PFK flux as shown in Figure 1D. Bistability is present for a wide range of K/P ratios. However, at a very high level of K/P ratios (>10), the bistable behavior disappears and the PFK flux exhibits a saturation type of kinetics. Thus, changes in the K/P ratio of the PFKFB result in the modulation of the steady state PFK flux.

Different isozymes of PFKFB have widely different K/P ratios [9] (Table 1), giving rise to different steady state behaviors of F6P-node. The brain isoform of PFKFB3 (with K/P∼700) will have a lower switch-up F6P concentration than the muscle isoform PFKFB1 (K/P∼0.4). In addition, hormonal or growth factor mediated regulations can modulate the K/P ratio of PFKFB isozymes (see review [16]). Such a regulation thus equips cells with an acute way to modulate the F6P-node steady state behavior, without undergoing a switch in their isozyme composition (chronic effect).

**Table 1:** Kinetic properties and the allosteric regulations of glycolytic enzymes expressed in mammalian cells.

Enzyme	Isozyme (Gene)	Isozyme (Protein Product)	Tissue Expression	Metabolite Regulation		References
				Inhibitors	Activators/ Substrates	
Phosphofructokinase (PFK)	*PFKM*	PFKM	Muscle	ATP	F16BP (Ka = 0.35 mM); F26BP; AMP; F6P	[12]
	*PFKL*	PFKL	Liver	ATP	F16BP (Ka = 0.65 mM); F26BP; AMP; F6P	[12], [14]
	*PFKP*	PFKP	Platelet	ATP	F26BP; AMP; F6P	[12]
Pyruvate Kinase (PK)	*PKLR*	PKL	Liver; Pancreatic Islets; Kidney	ATP (Ki = 0.05mM)	F16BP (Ka = 0.01 mM); PEP (Km = 0.6 mM)	[11]
		PKR	Erythrocytes	ATP (Ki = 0.12mM)	F16BP (Ka = 0.04 mM); PEP (Km = 1.2 mM)	[11]
	*PKM*	PKM1	Muscle; Heart; Brain	ATP (Ki = 2.5mM)	PEP (Km = 0.08 mM)	[11]
		PKM2	Fetal; Tumor Cells; Cultured Cells	ATP (Ki = 3.5mM)	F16BP (Ka = 0.04 mM); PEP (Km = 0.4 mM)	[11]
6-Phosphofructo-2-kinase/fructose-2,6-bisphosphatase (PFKFB)	*PFKFB1*	Liver	Liver (K/P = 1.5–2.5)	glucagon (PKA), PEP	X5P (glucose induced effect)	[9], [19], [61]
		Muscle	Muscle (K/P = 0.4)	PEP	insulin	[9], [19], [61]
	*PFKFB2*	H1-H4	Heart (K/P = 1.8)	PEP	AKT/PKB; PKA; PKC; AMPK	[9], [19], [61]
	*PFKFB3*	Ubiquitous	Brain; Placenta (K/P = 3.1)	PEP	-	[9], [19], [61]
		Inducible	Tumor (K/P = 710)	PEP	AMPK	[9], [19], [61]
	*PFKFB4*	T	Testis (K/P = 4.1)	PEP	-	[9], [19], [61]

### Bistable Behavior in Glycolysis

In the following discussions, we extend our analysis to the entire glycolysis pathway. For the purpose of this study, we will be focusing on the regulations of PFK, PFKFB and PK. Feedback inhibition of HK by G6P and feed-forward activation of PFK by F26BP were considered and kept active in all the simulations discussed later. The known regulations for PFK, PFKFB and PK can be grouped into two regulatory loops: Loop 1 and Loop 2. Loop 1 consists of the feedback activation of PFK by F16BP and activation of PFK by F26BP. Loop 2 encompasses three regulations consisting of the feed-forward activation of PK by F16BP, the feed-back inhibition of PFKFB by PEP and activation of PFK by F26BP. The three allosteric regulations in Loop 2, when active simultaneously, yield feedback activation effect on the activity of the glycolysis pathway.

The presence or absence of the allosteric regulations considered in Loop 1 and Loop 2 is determined by the isozymes that constitute the pathway. Loop 1 is operational with the muscle or liver isoforms of PFK (PFKM or PFKL), but not with the platelet isoform (PFKP) as the latter lacks the feedback activation by F16BP. Loop 2 is mainly seen with the expression of liver (PKL) or the red blood cell (PKR) or the M2 isoform of PK (PKM2) which are strongly activated by F16BP, but not with the M1 isoform (PKM1). Depending on the make-up of the isozymes, the glycolysis pathway in a tissue or a cell may have allosteric regulations of Loop 1, Loop 2, both or neither. This is illustrated in the expression profile of glycolytic isozymes compiled from published RNAseq transcriptome data of developing human embryos [41], HeLa cells [42], mouse adult tissues and transformed cells [43] (Tables S1 and S2).

We will show that the bistable behavior imparted by the allosteric regulation at F6P-node is extended to glycolysis pathways through Loop 1. Subsequently, we will also show that the allosteric regulation of Loop 2 also gives rise to bistability. However, Loop 2 encompasses a larger segment of glycolysis. Therefore the bistable behavior of Loop 2 will only be shown as glycolysis flux, rather than as an isolated node.

**Figure 2:**
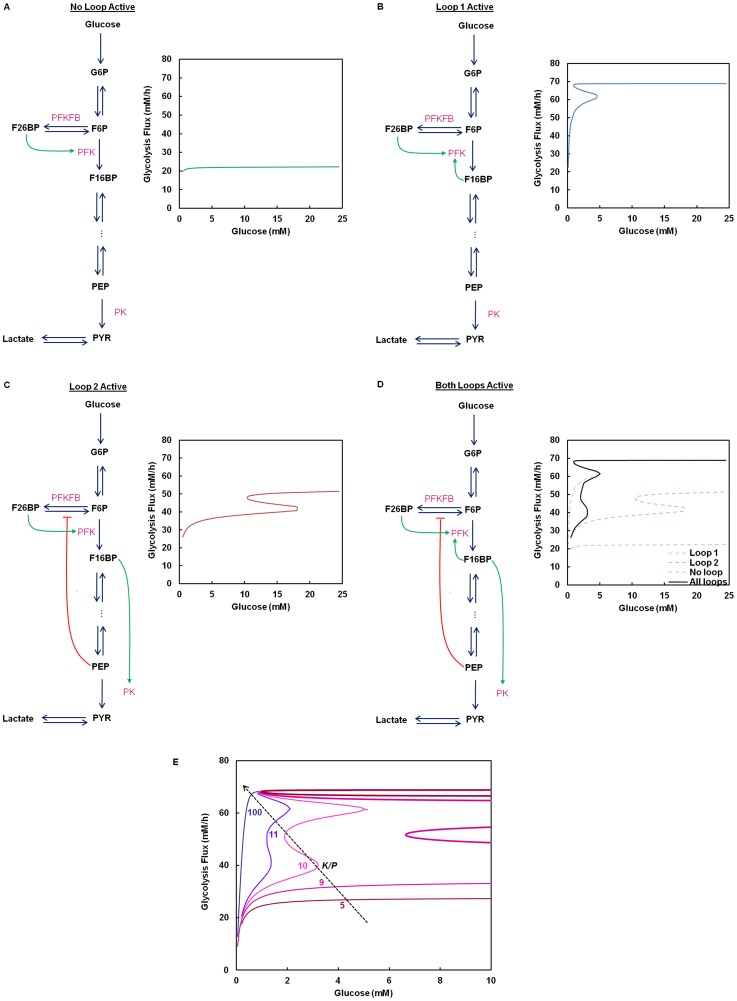
Multiplicity of steady states in the glycolysis flux. (**A**) Steady state glycolysis flux with neither Loop 1 nor Loop 2 active. The isozyme set consisting of PFKP (no activation by F16BP) and PKM1 (no activation by F16BP) were used in the simulation. The steady state flux exhibits classical Michaelis-Menten kinetics. (**B**) Steady state glycolysis flux with only Loop 1 active. When only Loop 1 is active, the glycolysis flux exhibits bistability. The isozyme set PFKL (***K_PFK,fbp_***  = 0.65 mM) and PKM1 were used in the simulation. (**C**) Steady state glycolysis flux with only Loop 2 active. When only Loop 2 is active, the glycolysis flux also exhibits bistability. The isozyme set PFKP and PKM2 (***K_PK,fbp_***  = 0.04 mM) were used in the simulation. (**D**) Steady state glycolysis flux with both Loop 1 and Loop 2 active. The isozyme set PFKL and PKM2 were used in the simulation. When both Loop 1 and Loop 2 are active, multiplicity of steady state resembling superposition of (**B**) and (**C**) is observed. For **A**–**D** the K/P of PFKFB was set at 10. (**E**) The effect of K/P modulation on the multiplicity of steady state in glycolysis. In this simulation PFKL and PKM2 were used, while K/P was varied.

The glycolysis flux with neither Loop 1 nor Loop 2 regulations is shown in Figure 2A. This case reflects a combination of PFKP (thus Loop 1 inoperative) and PKM1 (Loop 2 inactive), such as in oocyte or zygote in which PFKP and PKM1 are the dominant isozymes expressed (Table S1). The simulation was performed by omitting both the terms corresponding to the F16BP feedback activation in the equation for PFK (***K_PFK,fbp_***) and the F16BP feed-forward activation in the equation for PK (***K_PK,fbp_***). In addition, the K/P ratio of PFKFB was set to 10. The simulated steady state glycolysis flux exhibits no bistability at any K/P ratio (Figure 2A and Figure S2); the steady state flux approaches its maximum level at a relatively low glucose concentration.

The case that only Loop 1 is active occurs when either PFKL or PFKM and PKM1 are the dominant isoforms expressed. Such a combination of isozymes is seen mainly in various sections of brain tissue including cerebellum, cortex and frontal lobe (Table S2). The simulation was performed by setting ***K_PFK,fbp_***  = 0.65 mM (for PFKL [12], [14]) and omitting the F16BP feed-forward activation term in the rate equation for PK. The K/P ratio of PFKFB was set to 10. All the enzyme levels (including both PFK and PK) and all other kinetic constants were kept at the same values as in Figure 2A. As shown in Figure 2B, incorporation of Loop 1 (which is equivalent to introducing the F6P-node to glycolysis), elevates the glycolysis flux to a much higher level and a region of bistability is seen.

Similarly, the effect of the Loop 2 alone is shown in Figure 2C. This is the case when PFKP is the dominating PFK isozyme with PKL, PKR or PKM2 as the PK isozyme. Large and small intestine express such a combination of isozymes (Table S2). The simulation was performed by omitting the F16BP feedback activation term in the equation for PFK and setting ***K_PK,fbp_***  = 0.04 mM (for PKM2 [11]). The K/P ratio of PFKFB was set to 10. All the enzyme levels (including both PFK and PK) and all other kinetic constants were kept at the same values as in Figures 2A-B. In this case the extent of the maximal flux is somewhat lower than the case of Loop 1 alone. A bistable region is seen with both the switch-up and the switch-down concentrations of glucose shifted toward a higher level of glucose.

When both the loops are active, such as in case of hESCs, HeLa cells (Table S1) and mouse transformed cell lines (MEL and 10T1/2) (Table S2), the glycolysis flux has multi-stable behavior (Figure 2D). The simulation was performed by setting ***K_PFK,fbp_***  = 0.65 mM (for PFKL) and ***K_PK,fbp_***  = 0.04 mM (for PKM2). The K/P ratio of PFKFB was set to 10. All the enzyme levels (including both PFK and PK) and all other kinetic constants were kept at the same values as in Figures 2A–C. Three stable steady states and two unstable steady states can be seen for this case. The maximum flux is the same as that of Loop 1 alone while the multiple steady state region resembles the composite of the stability curves for Loop 1 and Loop 2 (Figures 2B and 2C).

The results show that the steady state glycolytic flux may be at a high flux or a low flux state depending on the presence or absence of the regulatory Loop 1 and Loop 2. Without Loop 1 and Loop 2 active, in the physiological range of glucose concentration (5–10 mM) glycolysis flux is at a low flux state (Figure 2A). With Loop 1 active alone, it will be at a high flux state (Figure 2B). To switch to a low flux state from a high flux state, the glucose concentration will need to decrease to a low level that is rarely seen physiologically; whereas if the initial flux is at a low state, as soon as the glucose level reaches a physiological level of 5 mM the flux will switch to a high state. The steady state behavior with Loop 2 active alone is rather different from the case of Loop 1 active alone; the flux in the physiological glucose concentration range is at a low state with a switch up concentration at the high end edge of physiological concentration (Figure 2C). Even if the flux is initially at a high state, it will switch to a low state when glucose decreases to a physiological level below 10 mM.

With both Loop 1 and Loop 2 active, the flux is at a high state in the physiological range of glucose. Interestingly, from a high flux state the system can switch only to the low state, bypassing the intermediate stable steady states. The intermediate stable steady states can be reached only from a low flux state by increasing glucose concentration. The stability of those steady states was verified by eigenvalue analysis.

In both the loops described above, F26BP plays a regulatory role through its allosteric control over PFK. The level of F26BP can be modulated by the K/P ratio of PFKFB. We have shown above that modulation of K/P affects the bistability behavior in the F6P-node alone (Figure 1D). This effect of flux modulation through K/P is also translated to the entire glycolysis flux (Figure 2E). The simulations were performed using exactly the same conditions as those used in Figure 2D, except for the K/P value which was varied. The multiple steady state region shifts as K/P ratio of PFKFB changes. At higher values the multiple steady state region is lost and the flux behaves like typical saturation type of kinetics. Reducing K/P has the effect of shifting the multiple steady state region to higher concentration range of glucose. At very low levels of K/P the multiple steady state region moves outside of the normal physiological range of glucose (>10 mM).

To evaluate the robustness of the multiple steady state behavior, sensitivity analysis on the multiplicity of steady states was performed by changing the levels of each enzyme (over a range of two orders of magnitude) while holding all the other kinetic parameter values constant. The results show that for all enzymes, the multiplicity of steady states can be seen to exist over a wide range of enzyme levels except for HK (Figure S3). It has been reported that such a tight control of HK, either at enzyme level or through allosteric regulation, is required in order for the glycolysis flux to reach a steady state [44]. Changing the concentration of the enzyme in the ranges shown in Figure S3 maintains the presence of the bistable behavior but shifts the switch-up and the switch-down concentrations.

We further examined the sensitivity of the steady state behavior to a number of model parameters whose value was kept constant in this study, including the cellular redox state ([NAD]/[NADH]), the ratio of mitochondrial pyruvate and lactate concentrations ([PYR]_m_/[LAC]) and the ratio of mitochondrial pyruvate and alanine concentrations ([PYR]_m_/[ALA]). For [NAD]/[NADH], multiplicity of steady states was observed over a wide range of the ratio (0.5 to 700) (Figure S4A). At very low [NAD]/[NADH] ratios (≤1), glycolytic flux has three steady states as compared to five steady states seen at higher [NAD]/[NADH] ratios (≥9). The data suggest that [NAD]/[NADH] ratio has a marginal effect at very low values. The effects of [PYR]_m_/[LAC] and [PYR]_m_/[ALA] were probed by maintaining [PYR]_m_ constant (at 0.1 mM) and varying either [LAC] or [ALA] (Figure S4B and S4C). Multiplicity of steady states was observed across the range in which lactate and alanine were varied. No effect on the steady state flux profile was observed when lactate was varied. Whereas, at higher concentrations of alanine, the switch-up concentration moves closer to upper bounds of (or in some cases beyond) the typical range of physiological glucose concentrations.

### Effect of Isozyme Composition on Steady State Behavior of Glycolysis

Many isoforms of the same enzyme are expressed at different levels in the same tissue cell. The presence of different isoforms with different allosteric regulations makes the steady state flux behavior deviate from that seen with a single isoform. We evaluated the effect of the isoform mixtures on the glycolytic flux. In each case, the total level of the isoform mix for each enzymatic step was kept at the same level as the one used in the original model (with single isoform for each enzymatic step). The steady state behaviors of single PFK isoforms (PFKM, PFKL or PFKP) as well as mixed expression of these isoforms, in conjunction with PKM2 are shown in Figure S5. In all cases, the steady state behavior of the isoform mix takes the shape of that conferred by the mixture's dominant isoform. As shown in Figures 2A–D, the presence of only a single isoform in any active Loop precisely fixes the switch-up and the switch-down glucose concentrations. Interestingly, co-expression of the dominant isoform with smaller fractions of other isoforms allows for the movement of the switch-up and switch-down glucose concentrations within the physiological range, thus equipping cells with yet another way to control the span of the bistable region (Figures S5D–F).

### Bistability in Cultured Cells

We employed HeLa cells to examine the bistability in glycolysis flux. HeLa cells initially grown on microcarriers in high glucose (HG) medium were split into two cultures with either low glucose (LG) (0.6 mM) or high glucose (HG) (25 mM) (see Materials and Methods). The cell concentration was kept low such that glucose would not be depleted in the LG condition due to the uptake by the cells. Cells were then allowed to reach steady states in LG and HG conditions, which were the low flux state (0.05 mmol/10^9^ cells/h) and high flux state (0.31 mmol/10^9^ cells/h), respectively. Cells from both these conditions were then re-suspended in medium containing varying glucose concentrations and the specific rates of glucose consumption and lactate production were monitored.

**Figure 3:**
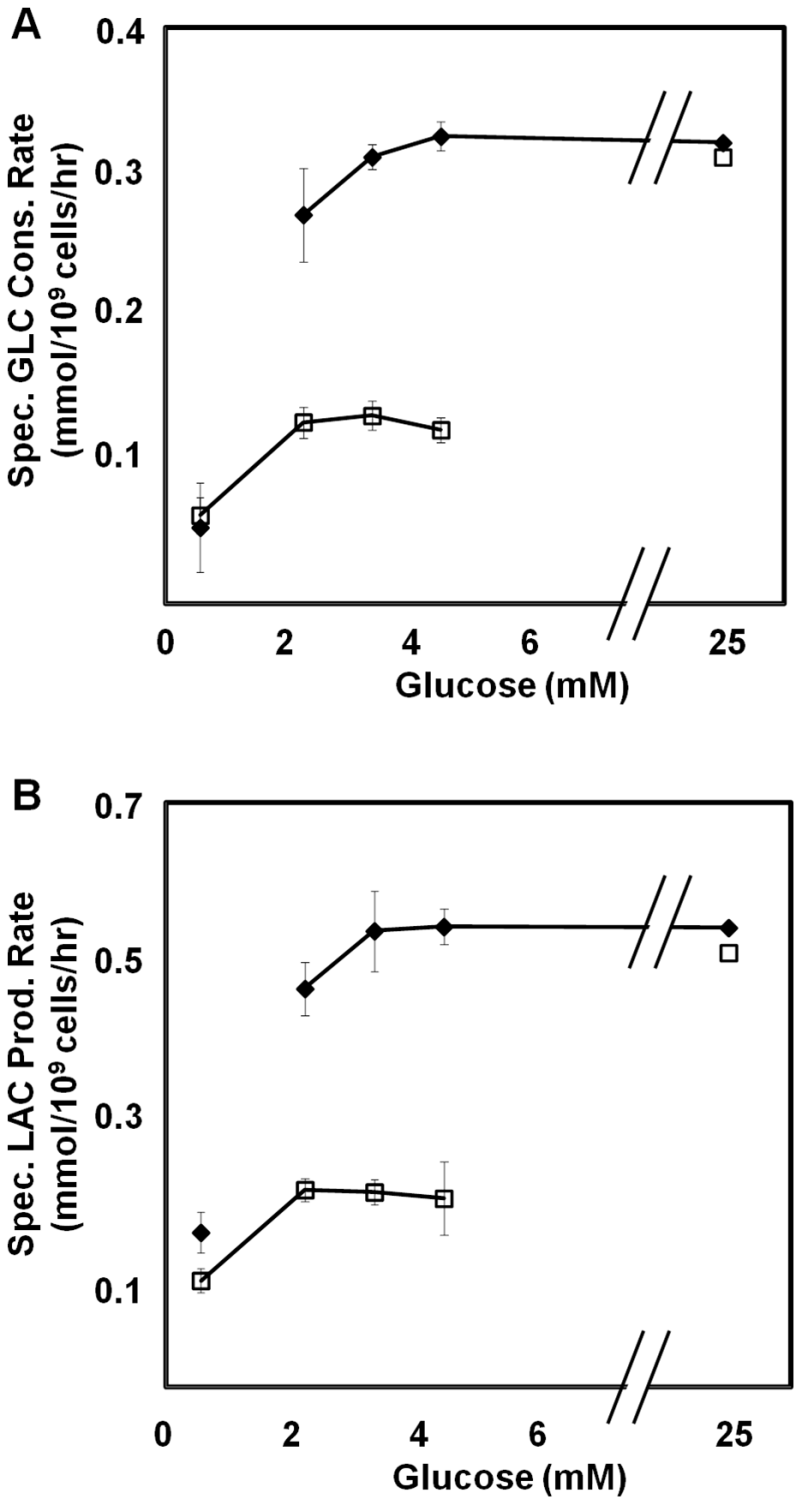
Bistability in cultured HeLa cells. Cells initially cultured in high glucose (♦) or low glucose (□) exhibit different rates of (**A**) specific glucose consumption and (**B**) specific lactate production when exposed to varying glucose concentrations.

When exposed to 25 mM glucose, cells showed high specific glucose consumption rate of ∼0.31 mmol/10^9^ cells/h regardless whether they were previously at a low flux or a high flux state (Figure 3A). When exposed to 0.6 mM glucose, cells from both HG and LG showed low specific glucose consumption rate of 0.05 mmol/10^9^ cells/h. However, when exposed to intermediate glucose concentrations of 2 mM, 3 mM and 4.5 mM, contrasting specific rates were observed for the cells from HG and LG conditions. Cells that were previously cultured in HG maintained high specific glucose consumption rate (0.31 mmol/10^9^ cells/h), whereas cells that were previously cultured in LG had low specific glucose consumption rate (0.05 mmol/10^9^ cells/h). The flux in the intermediate glucose concentration range is thus dependent on the prior state or history of the cell. Since a high flux of glucose is accompanied by a high lactate flux and vice versa, the bistable behavior of glucose flux should be reflected in lactate flux. This is indeed seen when lactate efflux was measured (Figure 3B).

The experimental results thus show the characteristic of bistability in HeLa cell glucose metabolism: cells which were at a high flux state (previously in HG) have to experience glucose levels below the “switch-down” concentration to reach the low flux state while cells from a low flux state (previously in LG) have to experience glucose levels higher than the “switch-up” concentration to reach the high flux state.

### PFKFB Modulates the Response Time of Metabolic State Switch

**Figure 4:**
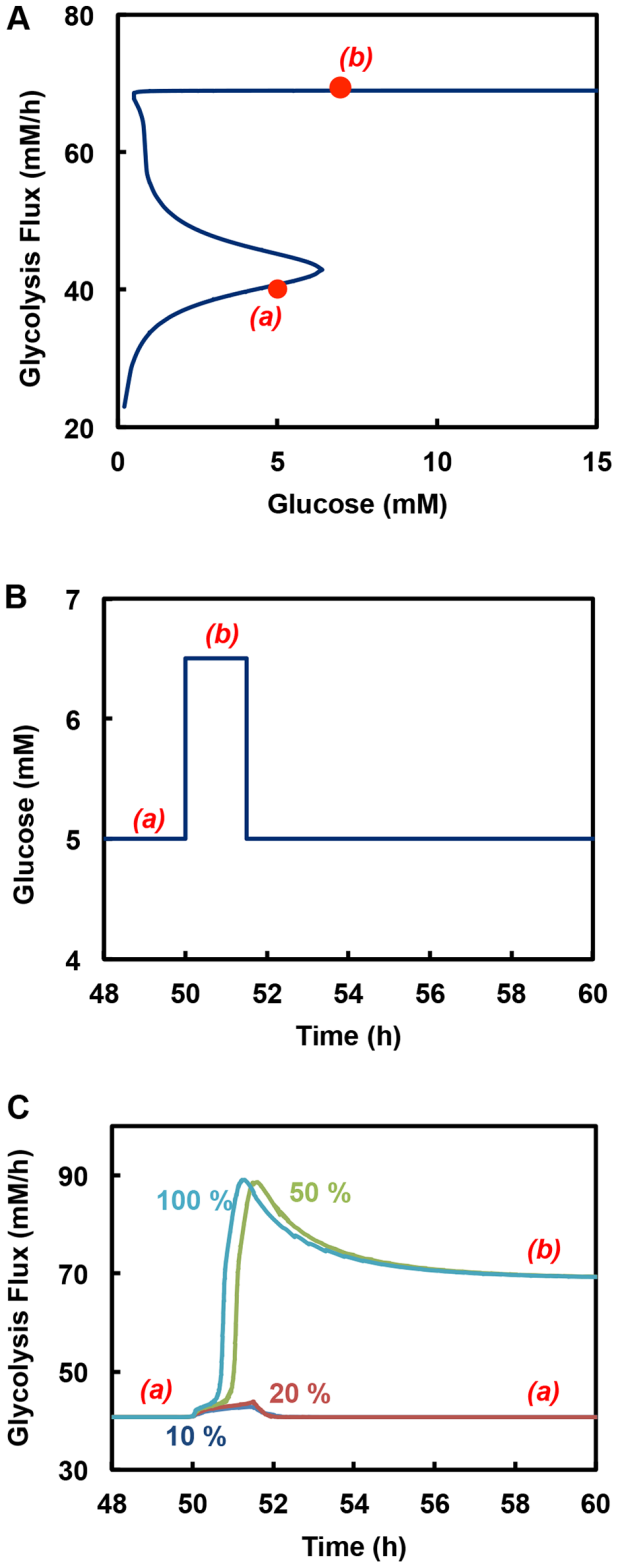
Effect of PFKFB levels on the transient response of glycolysis activity to pulse input in glucose concentration. (**A**) Steady state behavior of glycolysis at different levels of PFKFB. All steady state glycolytic plots for different PFKFB levels including 100%, 50%, 20% and 10% superimpose on each other. (**B**) Glucose pulse input. This figure shows the pulse input in glucose concentration made at 50h for duration of 1.5h and an amplitude sufficient enough to increase glucose concentration from low flux state *(a)* to high flux region *(b)*. (**C**) Response of the glycolysis to pulse input in glucose concentration for systems with different PFKFB levels. Systems were stationed at low flux state *(a)* when the pulse input was initiated. Systems with 100% and 50% PFKFB switch to the high state *(b)* in the given glucose pulse time. On the contrary, the response of the systems with 20% and 10% PFKFB is not fast enough to switch to the high state and therefore return to the low state when glucose is switched back to original concentration.

There exist four different isoforms of PFKFB with different K/P ratio (Table 1). Different isoform can thus confer different steady state glycolysis flux behavior (Figure 2E). Additionally the K/P ratio is subjected to hormonal regulation, providing a mechanism of rapid change in steady state behavior without resorting to synthesizing new isoform of PFKFB. However, since PFKFB catalyzes both the synthesis and hydrolysis of F26BP, the level of its expression does not alter the steady state level of F26BP, and does not have an effect on glycolysis flux, which has been confirmed by the use of the mathematical model (Figure 4A, glycolytic steady state plots with 100%, 50%, 20%, and 10% PFKFB superimpose on each other). Survey of PFKFB transcript level in different tissues reveals a wide range of expression (Figure S6). This diverse range of expression level, although does not change the steady state behavior, does have a profound effect on the dynamics.

The level of PFKFB affects the response time when glycolytic flux switches from a low state to a high state. This is illustrated by the simulated dynamic response of glycolytic flux upon increasing the glucose concentration corresponding to a low state to one at a high flux state. The K/P ratio of PFKFB used in the simulation is 10, corresponding to a condition in which a shift from a low flux state to a high flux state is possible in the physiological glucose concentration range. The system is initially at a low flux steady state in the bistable region. The glucose concentration is then increased to a level at which only high flux steady state is possible (Figure 4A). Given sufficient amount of time, the system will settle at a new high flux state. However, if the glucose concentration is switched back to the original level in the bistable region after a brief period of time (Figure 4B), two outcomes are possible: the system may remain at the high flux steady state, or returns to the low flux steady state.

With a high PFKFB level, the flux changes faster to the higher flux state and settles at a new steady state before the glucose concentration is returned to the original level in the bistable region (Figure 4C). It remains at the high flux state even after glucose is returned to the bistable region. In contrast, at a lower PFKFB level the response is sluggish when glucose concentration is given a step change. Upon switching glucose concentration to the original level, the flux returns to the original low flux state (Figure 4C). The response time to a switch to the new high flux steady state is clearly dependent on the level of PFKFB.

## Discussion

In this study we demonstrate that in the physiological range of glucose concentration the glycolysis flux exhibits multiple steady state behavior. The multiple steady states arise from the regulatory loops centering at PFK and PK. The presence of M/L isoforms PFK and the M2 isoform of PK, which are all subjected to activation by their respective effectors, give rise to steady state multiplicity.

**Figure 5:**
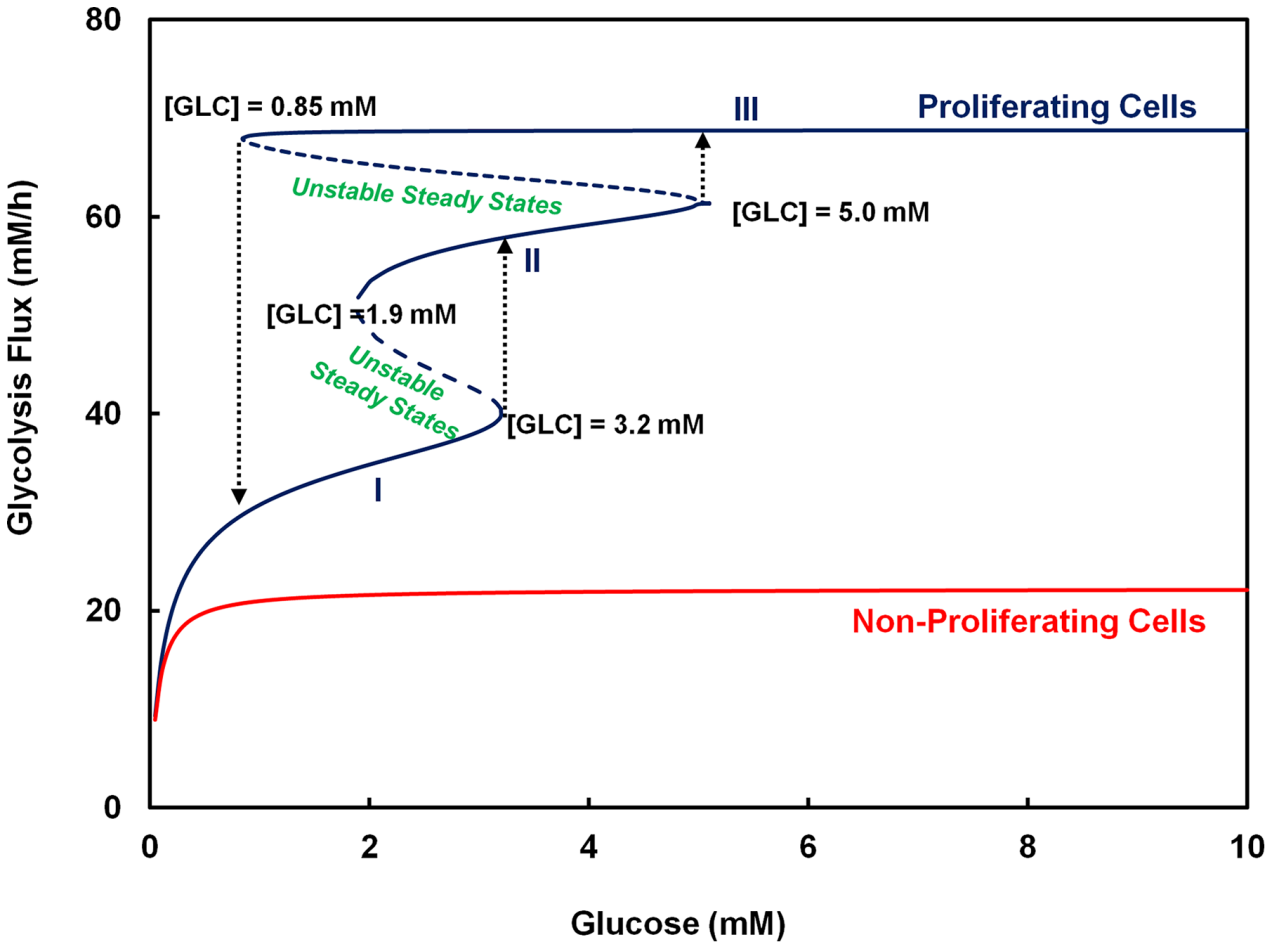
Glycolytic behaviors observed in mammalian cells. Two types of glycolytic steady state kinetics were observed. These include steady state behavior with no multiplicity of states or those with multiplicity of states. The type of glycolytic isozymes expressed forms the basis for presence or absence of multiplicity of states in glycolysis activity. In case of non-proliferating cell, isozyme combination comprising of PFKP, PKM1 and PFKFB with K/P  = 0.5 was used whereas in case of proliferating cell, isozyme combination including PFKM, PKM2 and PFKFB with K/P  = 10 was employed.

The sets of isoforms, (PFKM/PFKL and PKM2) and (PFKP and PKM1), confer contrasting multiple steady state and the saturation type steady state behaviors to glycolysis flux, respectively (Figure 5). With the latter set of isozymes, the flux is at a low flux state under physiological glucose concentrations. In contrast, the bistable region conferred by the former set of isozymes can span the physiological concentration range of glucose. The model thus predicts that the flux will be at a high flux state under physiological conditions. Only upon a long period of exposure to a very low glucose concentration (<∼1 mM) will the glyolysis flux switch to a low flux state. A high flux state is also associated with a high lactate production as shown by the model simulation (Figure S7).

In the bistable region, the stable steady state at which the glycolysis resides at a particular glucose concentration is dictated by the trajectory along which the system moves. Cells which are originally at a high flux state will remain at high flux state until the glucose concentration is reduced to a level below the switch-down concentration; whereas cells which are originally at a low flux state, will stay at low flux state until the glucose concentration is increased beyond the switch-up concentration. Using HeLa cells we demonstrated this effect of cell's “history” in terms of the fluxes of both glucose consumption and lactate production (Figure 3).

Metabolic switch between low and high flux states to meet the changing bioenergetic demands caused by oncogenic or developmental events may be brought about by expression of a different set of isozymes (see review [2], [3], [7]). Mature oocyte and zygote exhibit the oxidative type of metabolism [2]. As zygote differentiates and reaches the late blastocyst state, the metabolic phenotype changes to predominantly glycolytic. Such a change in the metabolic phenotype from the oxidative state of quiescent oocyte and zygote to glycolytic state of the blastocyst is associated with changes in glycolytic isozymes from PKM1, PFKP and PFKFB2 to PKM2, PFKM/PFKL and PFKFB3/PFKFB4 isozymes (Table S1). The concurrent switch in the isozyme pattern and the flux behavior observed in the above scenario matches that presented by our model simulations (Figure 5).

It should be noted that most cells express a mixture of isoforms rather than a single one [45]–[50]. Such pattern is also observed in the tissues described above (oocyte, zygote and blastocyst), where other isoforms are also expressed in small fractions (Table S1). We evaluated the effect of mixed expression of multiple isoforms of PFK on the multiple steady state behavior. When different isoforms of PFK are expressed, the isoforms which exhibit bistable behavior, namely PFKL and PFKM, dominate over PFKP unless PFKP is present in a much larger proportion than the other isoforms (Figure S5). It is also worth noting that not all proliferating cells and quiescent cells have the same isozyme patterns. However, it has been reported that fast proliferating cells typically express PFKM/PFKL, PKM2 and PFKFB3 as the major isoforms; whereas quiescent cells favor PFKP, PKM1 and other isoforms of PFKFB as the dominating isoforms [28], [51], [52].

Metabolic shift may also come about by hormonal or signaling regulation, such as those that change the K/P ratio of PFKFB (Figure 2E). For example, the K/P ratio of PFKFB in hepatocytes can be quickly modulated by glucagon-triggered cAMP signaling [9]. Furthermore, a number of factors that affect the allosteric state of PKM2 isozyme, including serine, SAICAR and phenylalanine, can influence the transition between the two metabolic states [20]–[22], [53].

PFKFB affects the activity of PFK through its reaction product F26BP. Different isoforms of PFKFB have different K/P ratios that give rise to different steady state behavior of glycolysis (Figure 2E). An isoform with a high K/P ratio yields a higher level of F26BP at steady state, exerts a stronger activation of PFK and moves the switch-up glucose concentration to lower levels. With hormonal actions that change the K/P of PFKFB, the steady state behavior of glycolysis can be altered quickly without resorting to changes in the isoform expression. Since PFKFB catalyzes both forward and reverse reaction of F26BP synthesis, the level of its expression does not alter the steady state level of F26BP, and does not have an effect on glycolysis flux. But the response time to reach a new steady state upon a change in glucose concentration is affected by the level of PFKFB expression (Figure 4).

In a separate study, an extended version of the current mathematical model has been used to examine the steady state behavior in glycolysis, in glucose and lactate concentration ranges that are beyond the physiological level but is of interest to industrial bioprocessing for pharmaceutical biologics production (Mulukutla et al., in preparation). In those cases, the high levels of lactate accumulated in culture causes inhibition of PFK [54], [55] and induces a shift in the metabolism to lactate consumption (Mulukutla et al., in preparation). The extended model includes the pentose phosphate pathway (PPP) and the TCA cycle. We observed that the extended model also showed multiple steady state behavior in glycolytic flux in the similar glucose concentration range and such complex glycolytic behavior also affect the dynamics of TCA cycle and PPP flux.

The mechanisms used to describe the enzyme reactions involved in the pathway and the values of the kinetic parameters used were all taken from the reported literature. The transcriptome data of mouse myeloma cells [37] were employed to estimate the relative abundance level of glycolysis isozymes. A survey of the archived transcriptome data of different tissue and cultured cells revealed a wide range of transcript level and proportion of isoforms for virtually all enzymes in glycolysis [56]. Cells of different types and arising from different tissues thus possess a varying steady state behavior. Exhaustive simulation for evaluation of steady state behavior on all possible enzyme combinations is clearly not feasible. We evaluated the range of enzyme expression level that gives multiple steady state behavior. By varying the concentration of one enzyme while keeping all other variables constant we show that steady state multiplicity is seen in a wide range of concentration of the two pivotal enzymes, PFK and PK (Figure S3).

The bistable behavior in glycolysis confers robustness to the response of glycolysis to changes in glucose concentration; within the physiological range of glucose concentration a change in the flux state is not readily realized unless via a regulatory action, differentiation or oncogenic event. In recent years there has been increasing interest in developing treatment strategies of suppressing hyperactive cellular metabolism to control cancer cell growth or diabetes development. The glycolysis flux behavior revealed in this study may be exploited to negatively manipulate the metabolism of tumor cells by rendering Loop 1 or Loop 2 inoperative. Decreasing the activation of PFK by F16BP can make Loop 1 less active. Tumor cells typically express PKM2 as the dominant pyruvate kinase isozyme. Disrupting the allosteric regulations of PKM2 activity, such as by administering SAICAR [21], will make Loop 2 inactive. Further, suppressing the K/P ratio of PFKFB3 through expression of TIGAR [57] or by the use of small molecule modulators such as 3-(3-pyridinyl)-1-(4-pyridinyl)-2-propen-1-one (3PO) can make Loop 2 inoperative [58]. A mechanistic understanding of the regulation of glycolytic flux will have a positive impact on the advances of these metabolism-based therapeutic treatments.

## Materials and Methods

### Mathematical Model of Glycolysis

An ordinary differential equation (ODE) model that encompasses glucose metabolism from glucose uptake through glycolysis, lactate production, and pyruvate transport into mitochondria was constructed. The model considers mass balances for the 12 reaction intermediates of glycolysis (Information S1, Mathematical Model for Glycolysis Flux section). The mechanistic rate equations for enzyme reactions and the values of the kinetic parameters were all reported previously and are described in detail in the Rate Equation section of Information S1. The abbreviations of enzymes and reaction intermediates are listed in the Table S3. The levels of each enzyme (or alternatively the V_max_) used in the ODE model are included in the Rate Equation section of Information S1.

### Steady State Solution of the Model

An algebraic model consisting of the steady state mass balance equations for the intermediates of all the reactions considered was derived from the ODE model. The algebraic model was used to evaluate all the possible steady states and the corresponding eigenvalues (Information S1). The solutions were obtained using the inbuilt numerical solver *fsolve* in Matlab (Mathworks, Inc.) computing environment. For each glucose concentration, positive and real-valued solutions were calculated using initial guesses of pseudorandom values drawn from the standard uniform distribution. Stability analysis was performed using eigenvalue analysis on each steady state solution obtained. In the simulations intracellular concentrations of energy nucleotides (ATP, ADP, AMP, NAD^+^, NADH), metal ions influencing the kinetics of glycolytic enzymes (Mg^2+^, K^+^, Ca^2+^ etc) and several metabolic intermediates (mitochondrial pyruvate and extracellular lactate) were set to be constant in order to insulate glycolysis behavior from the effect of their concentration fluctuations. The fixed concentrations of these are tabulated in Table S4.

### Model Transient Simulations

Transient simulations of the ODE model were performed in the Matlab (Mathworks, Inc.) computing environment using the implicit numerical ODE solver *ode15*. The inputs for the model were the concentrations of glucose and lactate. A simulation time of 500 time steps (hour) was used to ensure the steady state was reached. The intracellular concentrations of energy nucleotides (ATP, ADP, AMP, NAD^+^, NADH), and metal ions influencing the kinetics of glycolytic enzymes (Mg^2+^, K^+^, Ca^2+^ etc) and several metabolic intermediates (mitochondrial pyruvate and extracellular lactate) were set to be constant in order to insulate glycolysis behavior from the effect of their concentration fluctuations. Their concentrations are tabulated in Table S4.

### Cell Culture

HeLa cell line, originally obtained from ATCC (Manassas, VA) was a generous gift from Dr. Kim Do-Hyung and has been reported previously [59]. HeLa cells were cultured in DMEM medium (Invitrogen, 11995-065) supplemented with 4% fetal bovine serum at 37°C in 5% CO_2_. 100 mL culture with 5 g/L of Cytodex 1 microcarriers (GE Healthcare, 17-0448-01) was carried out in 250 mL spinner flasks. HeLa cells were inoculated at 2×10^5^ cells/mL on Day 0. The procedure for microcarrier culture has been described previously [60]. On Day 5, cells on microcarriers were washed and re-suspended at 1×10^5^ cells/mL in DMEM medium containing either 0.6 mM (LG) or 25 mM (HG) of glucose. The cell concentration was intentionally kept low such that the glucose concentration in the culture could be maintained at a relatively constant level without getting depleted. The two flasks were then maintained for a further 12 hours to allow the cells in LG condition to reach a low flux state and those in HG condition to reach a high flux state. Subsequently, the cells from each flask were washed twice with PBS and transferred to the wells of a 12-well plate containing 1 mL of medium with varying glucose concentration. Cells were inoculated at a high enough concentration (2×10^6^ cells/mL) to allow measurable changes in the medium glucose concentration due to cellular glucose uptake. Supernatants were collected at time 0, 2, 4, and 6 hours which were then assessed for glucose and lactate levels using the Infinity Glucose reagent (Thermo Scientific, TR15421) and YSI 2700 SELECT industrial analyzer (YSI Inc.), respectively. Subsequently, using linear regression, specific rates of glucose uptake and lactate production were calculated from the glucose/lactate measurement data.

## Supporting Information

Figure S1 **Steady state behavior of F6P-node with PFKP as the sole PFK isozyme expressed.** F6P-node was simulated using PFKP as the sole PFK isozyme, at different K/P ratios (range: 0.5–50). In all the cases, the steady state flux of the system (J_PFK_) followed the Michaelis-Menten type of kinetics. No multiplicity of states was observed in the range of K/P simulated.
(TIF)

Figure S2 **Steady state behavior of the glycolysis flux with no loop active and with PFKP as the sole PFK isozyme expressed.** The steady state glycolysis flux was simulated using PFKP as the sole PFK isozyme, at different K/P ratios (range: 5–100). In all the cases, the steady state flux followed the Michaelis-Menten type of kinetics. No multiplicity of states was observed in the range of K/P simulated.
(TIF)

Figure S3 **Bounds of enzyme activity within which bistability in glycolysis is observed.** Sensitivity analysis was performed on the glycolytic enzyme activity levels. Each enzyme level was varied individually while holding all other parameters constant. The values shown are normalized to the concentration of enzyme used in the original simulation (shown in Figure 2D). The range of enzyme activities in which bistability was observed for each enzyme are plotted. The majority of enzymes have a large range of enzyme activity in which bistable behavior is observed for complete glycolysis. Only few enzymes including HK, ALDO and GAPDH have small enzyme activity range for bistable behavior.
(TIF)

Figure S4 **Sensitivity of the steady state behavior of glycolysis to the perturbations in:** (A) NAD/NADH ratio (B) [Pyruvate]_m_/[Lactate] ratio and (C) [Pyruvate]_m_/[Alanine] ratio.
(TIF)

Figure S5 **Effect of single or mixtures of PFK isozymes on the bistability in glycolysis.** (**A**) Single PFKM isozyme (**B**) Single PFKL isozyme (**C**) Single PFKP isozyme (**D**) Mixtures of varying levels of PFKM and PFKP. (**E**) Mixtures of varying levels of PFKL and PFKP. (**F**) Mixtures of varying levels of PFKM and PFKL.
(TIF)

Figure S6 **PFKFB expression in various tissues.** Transcriptome data for mouse tissue were obtained from previously reported work (reference [57] of Information S1). Raw files were obtained from the NCBI GEO website with accession number GSE9954. The raw data were used to obtain the intensity data for all the probes and they were normalized by using linear normalization to a mean value of 500. The combined expression levels of PFKFB isozymes (PFKFB1-4) in those tissues were then plotted. Expression of PFKFB in muscle and adipose tissues was an order of magnitude different as compared to proliferating cells including embryonic stem cells (ES_cells) and fetus.
(TIF)

Figure S7 **Experimental data of glycolysis rate at varying glucose concentration.** Data from continuous culture of mouse hybridoma cells (reference [58] of Information S1) were used to plot the metabolic rates as a function of glucose concentration (**A**). The continuous culture data were from a total of 14 runs and reported data were all from steady states with a dilution rate (or growth rate) in the range of 0.30 to 0.33 h^−1^. (**B**) The ratio of lactate production (analogous to LDH rate) to glucose consumption (analogous to glycolysis rate) is shown as ΔL/ΔG. A sharp transition from high flux state to a low flux state can be seen (0.24 mM glucose). The overlapping region of high flux and low flux state resembles that of bistability (0.17–0.24 mM). The ΔL/ΔG plot is consistent with that postulated in Warburg effect. (**C**–**D**) Simulation results corresponding to the glycolysis activity and ΔL/ΔG shown in (**A**–**B**).
(TIF)

Table S1 **Composition of the transcript levels of several glycolysis isozymes at various stages of human embryonic development and cell lines.**
(DOCX)

Table S2 **Composition of the transcript levels of several glycolysis isozymes in various mouse organs and cell lines.**
(DOCX)

Table S3 **Nomenclature.**
(DOCX)

Table S4 **Fixed parameter values in the model.**
(DOCX)

Information S1 **Supplementary information.**
(DOCX)
