# Supplementary figures and images for: Bistability in Glycolysis Pathway as a Physiological Switch in Energy Metabolism
Source: PLoS One. 2014 Jun 9;9(6):e98756. doi: 10.1371/journal.pone.0098756 (PMC4049617; doi:10.1371/journal.pone.0098756)

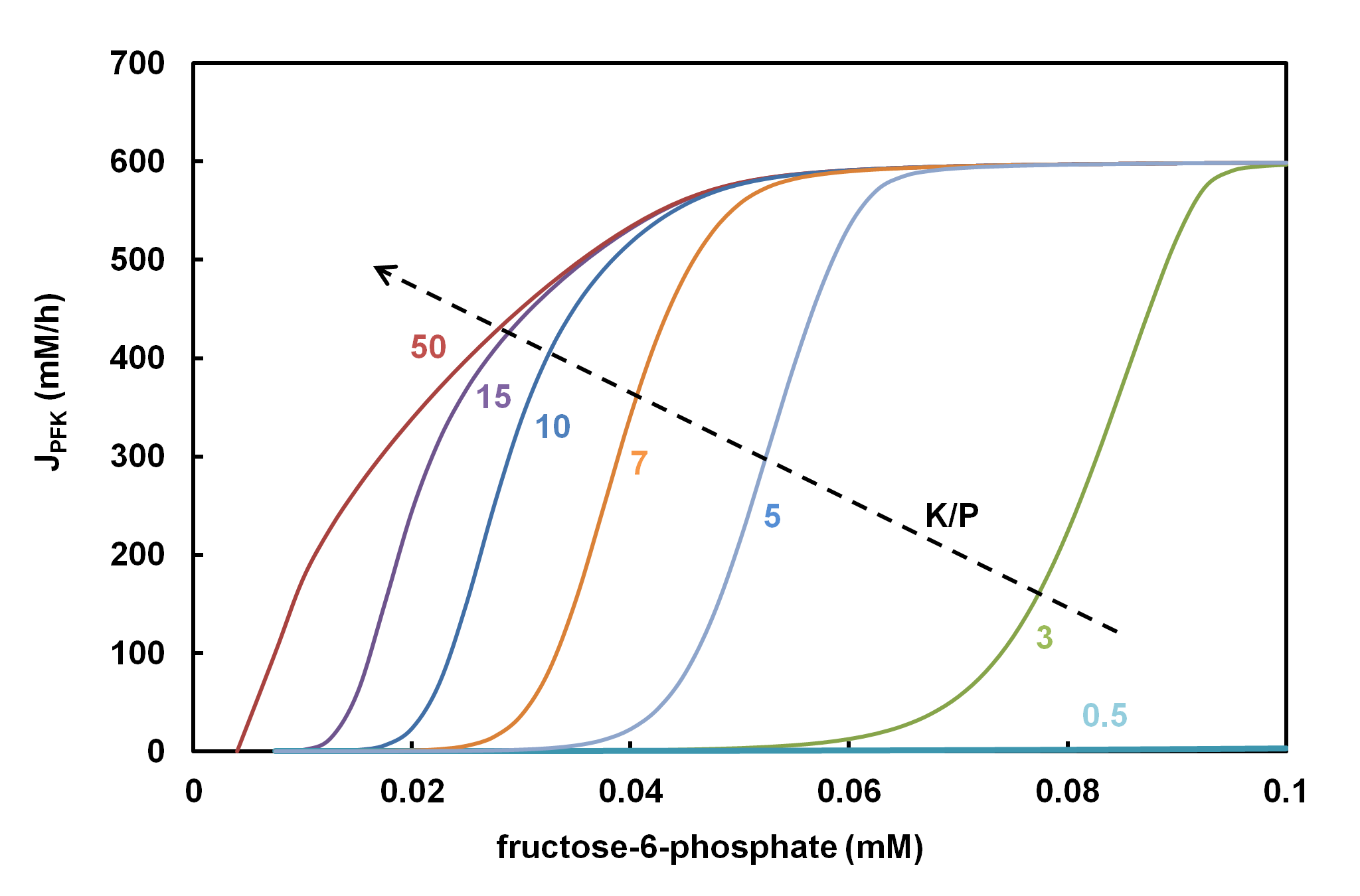

Supplement: Figure S1 — Steady state behavior of F6P-node with PFKP as the sole PFK isozyme expressed. F6P-node was simulated using PFKP as the sole PFK isozyme, at different K/P ratios (range: 0.5–50). In all the cases, the steady state flux of the system (JPFK) followed the Michaelis-Menten type of kinetics. No multiplicity of states was observed in the range of K/P simulated. (TIF) [file pone.0098756.s001.tif]

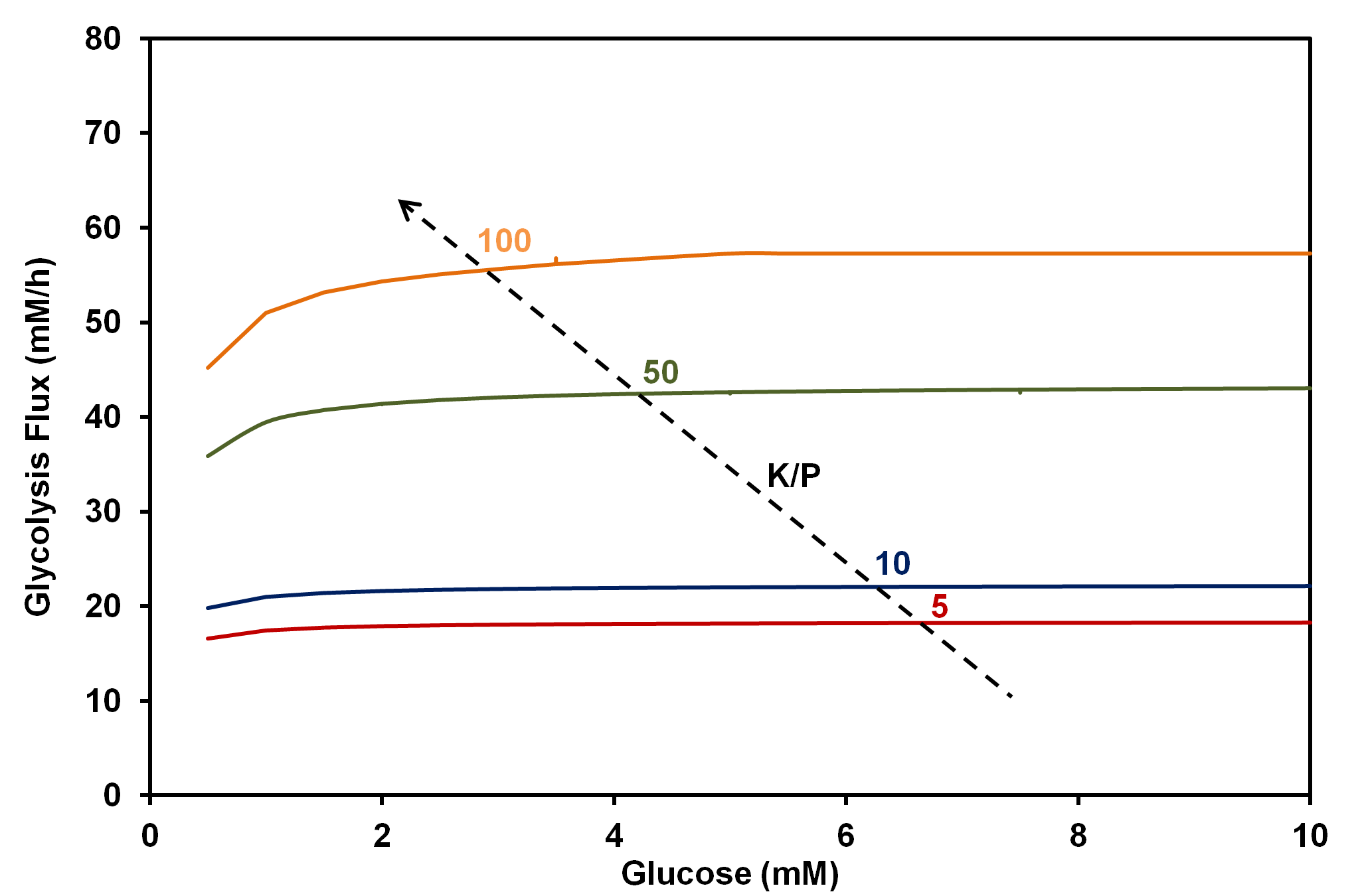

Supplement: Figure S2 — Steady state behavior of the glycolysis flux with no loop active and with PFKP as the sole PFK isozyme expressed. The steady state glycolysis flux was simulated using PFKP as the sole PFK isozyme, at different K/P ratios (range: 5–100). In all the cases, the steady state flux followed the Michaelis-Menten type of kinetics. No multiplicity of states was observed in the range of K/P simulated. (TIF) [file pone.0098756.s002.tif]

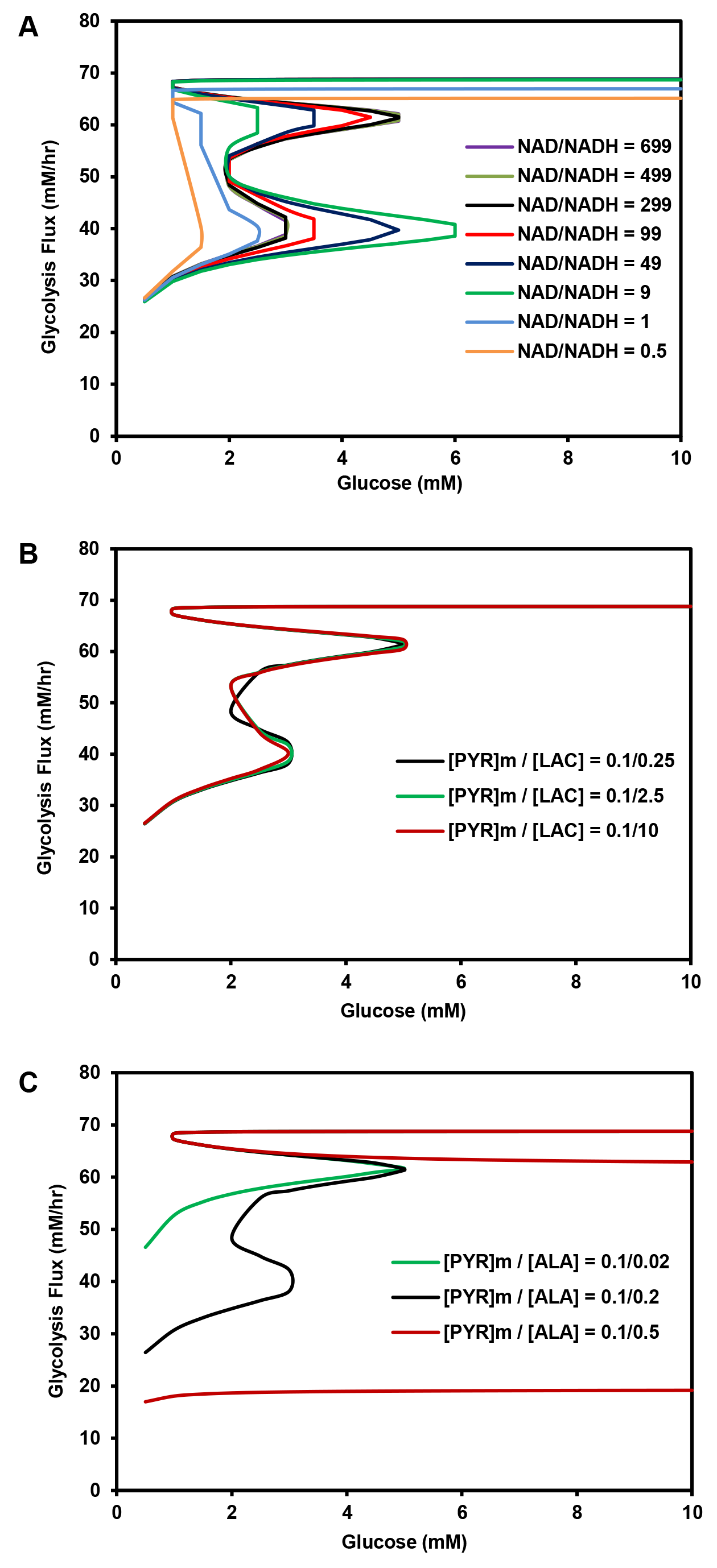

Supplement: Figure S4 — Sensitivity of the steady state behavior of glycolysis to the perturbations in: (A) NAD/NADH ratio (B) [Pyruvate]m/[Lactate] ratio and (C) [Pyruvate]m/[Alanine] ratio. (TIF) [file pone.0098756.s004.tif]

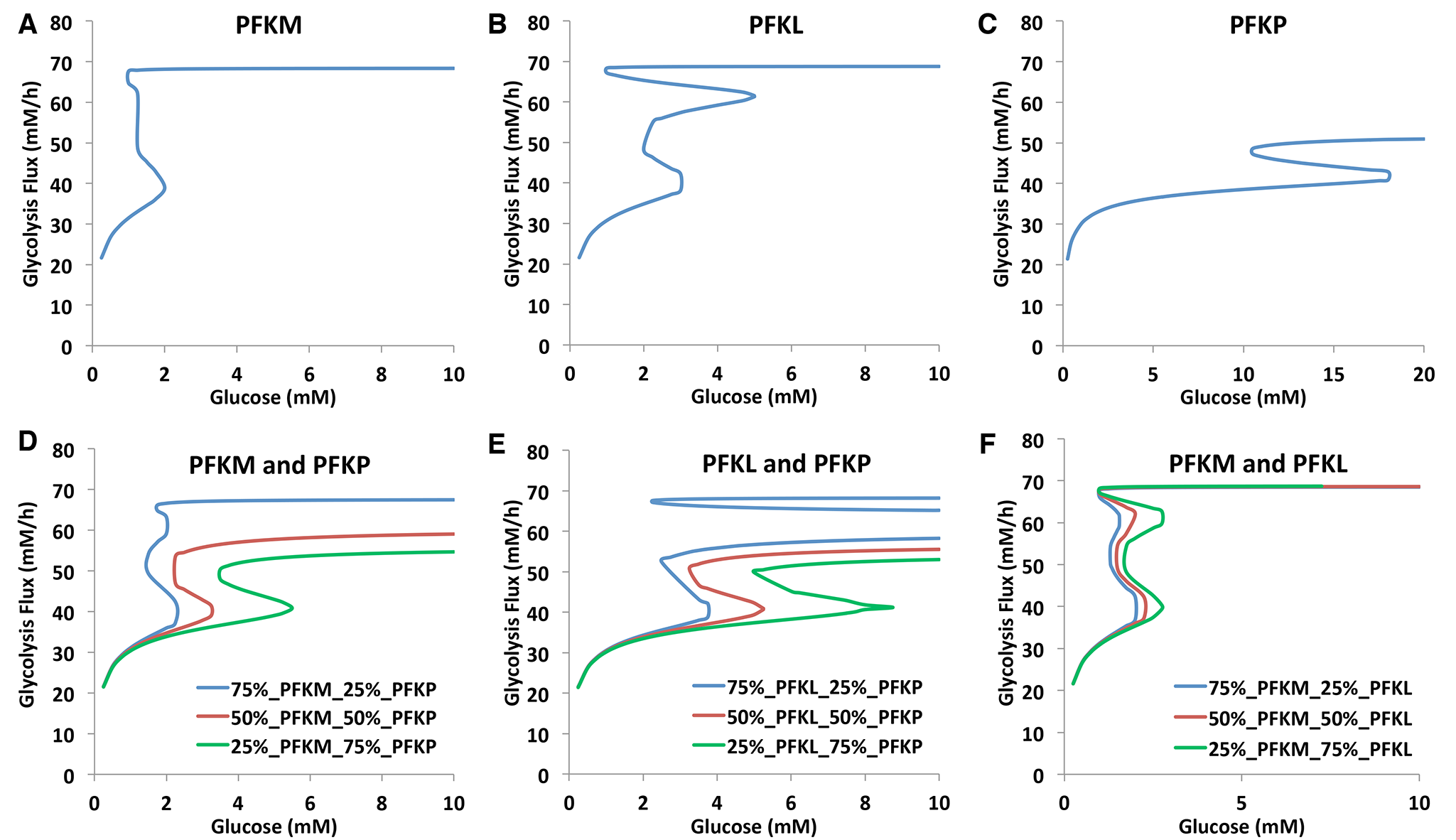

Supplement: Figure S5 — Effect of single or mixtures of PFK isozymes on the bistability in glycolysis. (A) Single PFKM isozyme (B) Single PFKL isozyme (C) Single PFKP isozyme (D) Mixtures of varying levels of PFKM and PFKP. (E) Mixtures of varying levels of PFKL and PFKP. (F) Mixtures of varying levels of PFKM and PFKL. (TIF) [file pone.0098756.s005.tif]

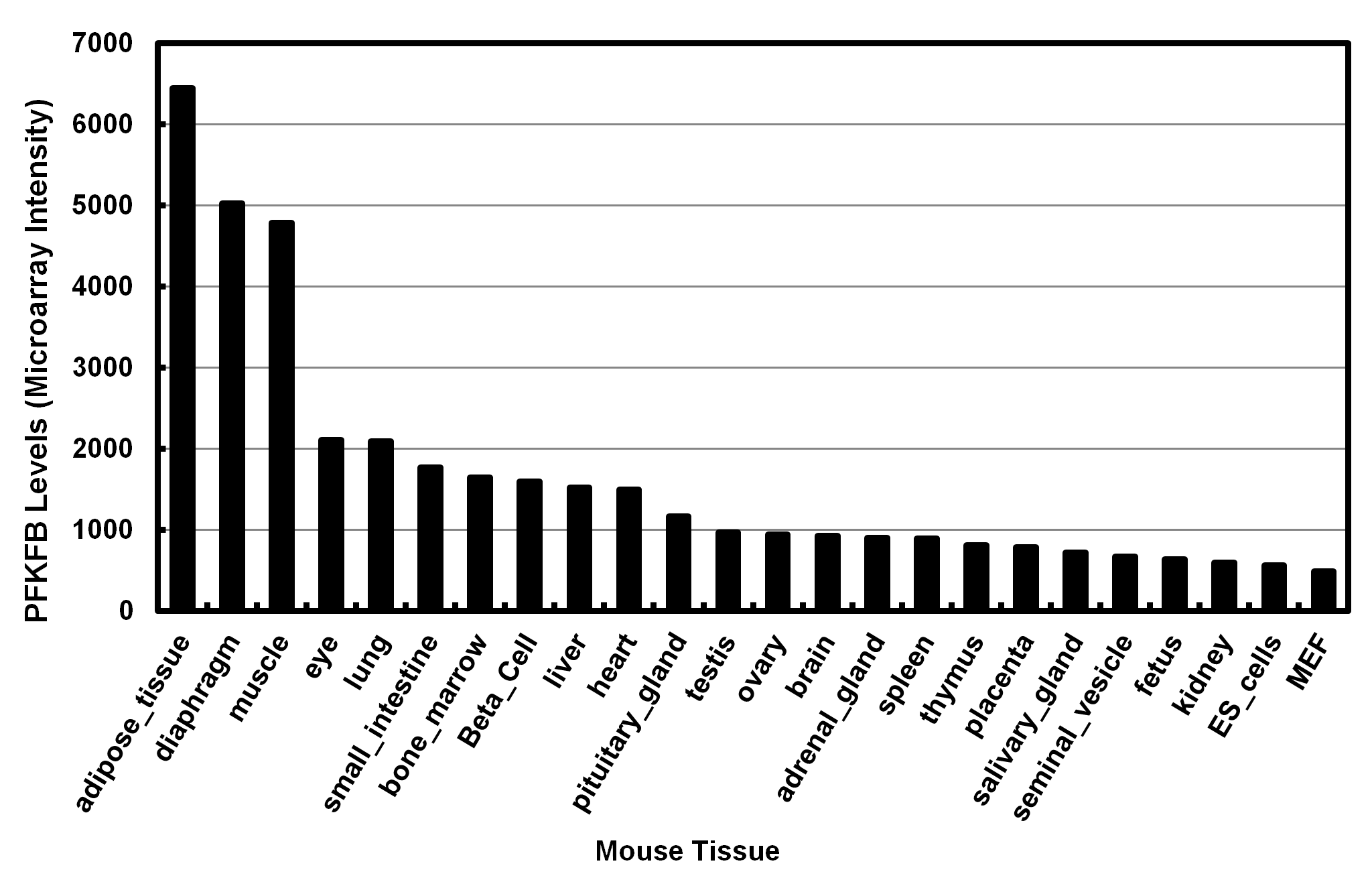

Supplement: Figure S6 — PFKFB expression in various tissues. Transcriptome data for mouse tissue were obtained from previously reported work (reference [57] of Information S1). Raw files were obtained from the NCBI GEO website with accession number GSE9954. The raw data were used to obtain the intensity data for all the probes and they were normalized by using linear normalization to a mean value of 500. The combined expression levels of PFKFB isozymes (PFKFB1-4) in those tissues were then plotted. Expression of PFKFB in muscle and adipose tissues was an order of magnitude different as compared to proliferating cells including embryonic stem cells (ES_cells) and fetus. (TIF) [file pone.0098756.s006.tif]

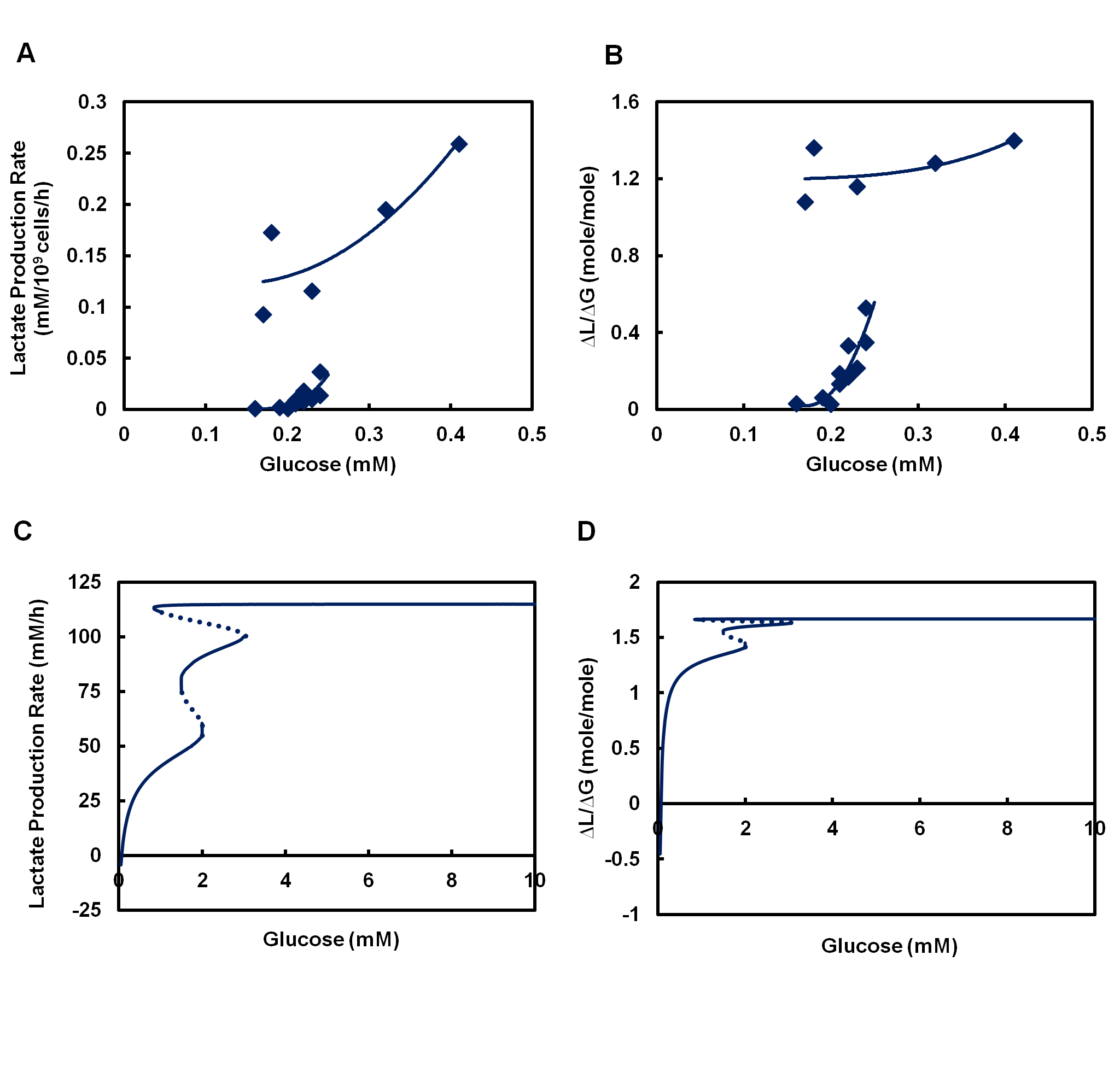

Supplement: Figure S7 — Experimental data of glycolysis rate at varying glucose concentration. Data from continuous culture of mouse hybridoma cells (reference [58] of Information S1) were used to plot the metabolic rates as a function of glucose concentration (A). The continuous culture data were from a total of 14 runs and reported data were all from steady states with a dilution rate (or growth rate) in the range of 0.30 to 0.33 h−1. (B) The ratio of lactate production (analogous to LDH rate) to glucose consumption (analogous to glycolysis rate) is shown as ΔL/ΔG. A sharp transition from high flux state to a low flux state can be seen (0.24 mM glucose). The overlapping region of high flux and low flux state resembles that of bistability (0.17–0.24 mM). The ΔL/ΔG plot is consistent with that postulated in Warburg effect. (C–D) Simulation results corresponding to the glycolysis activity and ΔL/ΔG shown in (A–B). (TIF) [file pone.0098756.s007.tif]
