# Supplementary material for: Bistability in Glycolysis Pathway as a Physiological Switch in Energy Metabolism
Source: PLoS One. 2014 Jun 9;9(6):e98756. doi: 10.1371/journal.pone.0098756 (PMC4049617; doi:10.1371/journal.pone.0098756)
Supplement: Table S1 — Composition of the transcript levels of several glycolysis isozymes at various stages of human embryonic development and cell lines. (DOCX) [file pone.0098756.s008.docx]

**Table S1.** Composition of the transcript levels of several glycolysis isozymes at various stages of human embryonic development and cell lines.

| **Enzyme** | **Isozyme** | **Oocyte** | **Zygote** | **2cell** | **4cell** | **8cell** | **Morula** | **Blastocyst** | **hESC p0** | **hESC p10** | **HeLa** |
| --- | --- | --- | --- | --- | --- | --- | --- | --- | --- | --- | --- |
|  |  |  |  |  |  |  |  |  |  |  |  |
| **Hexokinase (HK)** | **%HK1** | 0 | 0 | 0 | 6 | 10 | 19 | 15 | 73 | 80 | 69 |
|  | **%HK2** | 100 | 100 | 100 | 94 | 87 | 76 | 84 | 20 | 18 | 31 |
|  | **%HK3** | 0 | 0 | 0 | 0 | 0 | 0 | 0 | 0 | 0 | 0 |
|  | **%GCK** | 0 | 0 | 0 | 0 | 3 | 5 | 1 | 7 | 2 | 0 |
|  |  |  |  |  |  |  |  |  |  |  |  |
| **6-Phosphofructo-2-kinase/fructose-2,6-bisphosphatase (PFKFB)** | **%PFKFB1** | 9 | 2 | 0 | 0 | 0 | 0 | 0 | 0 | 6 | 0 |
|  | **%PFKFB2** | 65 | 57 | 78 | 69 | 11 | 22 | 7 | 7 | 20 | 57 |
|  | **%PFKFB3** | 9 | 7 | 7 | 9 | 87 | 65 | 0 | 4 | 12 | 17 |
|  | **%PFKFB4** | 16 | 34 | 15 | 22 | 2 | 13 | 93 | 89 | 62 | 27 |
|  |  |  |  |  |  |  |  |  |  |  |  |
| **Phosphofructokinase (PFK)** | **%PFKL** | 1 | 4 | 5 | 2 | 4 | 10 | 51 | 11 | 20 | 14 |
|  | **%PFKM** | 9 | 5 | 6 | 4 | 17 | 42 | 9 | 65 | 50 | 52 |
|  | **%PFKP** | 90 | 91 | 89 | 94 | 78 | 48 | 39 | 24 | 30 | 34 |
|  |  |  |  |  |  |  |  |  |  |  |  |
| **Pyruvate kinase (PK)** | **%PKL** | 0 | 0 | 0 | 0 | 0 | 0 | 0 | 0 | 0 | 0 |
|  | **%PKR** | 0 | 0 | 0 | 0 | 0 | 0 | 0 | 0 | 0 | 0 |
|  | **%PKM1** | 67 | 64 | 63 | 50 | 17 | 12 | 4 | 10 | 6 | 11 |
|  | **%PKM2** | 33 | 36 | 37 | 50 | 83 | 88 | 96 | 90 | 94 | 89 |
