# Supplementary material for: Bistability in Glycolysis Pathway as a Physiological Switch in Energy Metabolism
Source: PLoS One. 2014 Jun 9;9(6):e98756. doi: 10.1371/journal.pone.0098756 (PMC4049617; doi:10.1371/journal.pone.0098756)
Supplement: Table S2 — Composition of the transcript levels of several glycolysis isozymes in various mouse organs and cell lines. (DOCX) [file pone.0098756.s009.docx]

**Table S2.** Composition of the transcript levels of several glycolysis isozymes in various mouse organs and cell lines.

|  | **Bladder** | **Cerebellum** | **Cortex** | **Frontal Lobe** | **Colon** | **Heart** | **Large Intestine** | **Lung** | **Small Intestine** | **Spleen** | **Testis** | **Thymus** | **ES_Bruce4** | **ES_E14** | **MEL** | **10T1/2** |
| --- | --- | --- | --- | --- | --- | --- | --- | --- | --- | --- | --- | --- | --- | --- | --- | --- |
| **%PKM1** | 60 | 89 | 87 | 84 | 11 | 92 | 12 | 21 | 6 | 8 | 75 | 94 | 9 | 18 | 22 | 13 |
| **%PKM2** | 40 | 11 | 13 | 16 | 86 | 8 | 73 | 79 | 78 | 90 | 25 | 6 | 91 | 81 | 67 | 87 |
| **%PKL** | 0 | 0 | 0 | 0 | 2 | 0 | 14 | 0 | 15 | 1 | 0 | 0 | 0 | 0 | 5 | 0 |
| **%PKR** | 0 | 0 | 0 | 0 | 1 | 0 | 1 | 0 | 1 | 1 | 0 | 0 | 0 | 0 | 5 | 0 |
|  |  |  |  |  |  |  |  |  |  |  |  |  |  |  |  |  |
| **%PFKL** | 21 | 12 | 20 | 22 | 70 | 12 | 22 | 46 | 33 | 62 | 8 | 43 | 27 | 52 | 40 | 68 |
| **%PFKM** | 32 | 69 | 59 | 54 | 6 | 85 | 7 | 32 | 7 | 12 | 56 | 29 | 1 | 8 | 43 | 28 |
| **%PFKP** | 47 | 19 | 21 | 24 | 24 | 4 | 71 | 22 | 61 | 26 | 36 | 28 | 72 | 40 | 17 | 4 |
|  |  |  |  |  |  |  |  |  |  |  |  |  |  |  |  |  |
| **%PFKFB1** | 7 | 2 | 3 | 3 | 4 | 31 | 4 | 4 | 1 | 5 | 5 | 9 | 2 | 19 | 6 | 24 |
| **%PFKFB2** | 21 | 25 | 30 | 37 | 11 | 40 | 6 | 12 | 4 | 5 | 12 | 8 | 73 | 24 | 34 | 2 |
| **%PFKFB3** | 46 | 48 | 34 | 33 | 11 | 21 | 17 | 40 | 15 | 52 | 27 | 68 | 23 | 48 | 39 | 2 |
| **%PFKFB4** | 25 | 26 | 33 | 28 | 74 | 9 | 73 | 44 | 80 | 38 | 55 | 16 | 2 | 9 | 21 | 73 |
