# Supplementary material for: Bistability in Glycolysis Pathway as a Physiological Switch in Energy Metabolism
Source: PLoS One. 2014 Jun 9;9(6):e98756. doi: 10.1371/journal.pone.0098756 (PMC4049617; doi:10.1371/journal.pone.0098756)
Supplement: Table S3 — Nomenclature. (DOCX) [file pone.0098756.s010.docx]

**Table S3**. Nomenclature

| \| **Metabolite Symbol** \| **Name** \| \| --- \| --- \| \| GLC \| Glucose \| \| G6P \| Glucose-6-phosphate \| \| F6P \| Fructose-6-phosphate \| \| F16BP \| Fructose-1,6-bisphosphate \| \| F26BP \| Fructose-2,6-bisphosphate \| \| GAP \| Glyceraldehyde-3-phosphate \| \| DHAP \| Dihydroxyacetone phosphate \| \| 13BPG \| 1,3-bisphosphoglycerate \| \| 3PG \| 3-phosphoglycerate \| \| 2PG \| 2-phosphoglycerate \| \| PEP \| Phosphoenolpyruvate \| \| PYR \| Pyruvate \| \| LAC \| Lactate \| \| NADH \| Nicotinamide adenine dinucleotide (reduced) \| \| NAD \| Nicotinamide adenine dinucleotide (oxidized) \| \| NADPH \| Nicotinamide adenine dinucleotide phosphate (reduced) \| \| NADP \| Nicotinamide adenine dinucleotide phosphate (oxidized) \| |
| --- | --- | --- | --- | --- | --- | --- | --- | --- | --- | --- | --- | --- | --- | --- | --- | --- | --- | --- | --- | --- | --- | --- | --- | --- | --- | --- | --- | --- | --- | --- | --- | --- | --- | --- | --- | --- |
| \| **Enzyme Symbol** \| **Name** \| \| --- \| --- \| \| GLUT \| Glucose transporter \| \| HK \| Hexokinase \| \| GPI \| Glucose phosphate isomerase \| \| PFK \| Phosphofructokinase \| \| PFKFB \| 6-phosphofructo-2-kinase/fructose-2,6-bisphosphatase \| \| ALDO \| Aldolase \| \| TPI \| Triosephosphate isomerase \| \| GAPD \| Glyceraldehyde-3-phosphate dehydrogenase \| \| PGK \| Phosphoglycerate kinase \| \| PGM \| Phosphoglycerate mutase \| \| EN \| Enolase \| \| PK \| Pyruvate kinase \| \| LDH \| Lactate dehydrogenase \| \| PYRH \| Pyruvate mitochondrial transporter \| |
| \| **Subscript** \| **Compartment** \| \| --- \| --- \| \| e \| Extracellular \| \| c \| Cytosol \| \| m \| Mitochondria \| |
