# Supplementary material for: Bistability in Glycolysis Pathway as a Physiological Switch in Energy Metabolism
Source: PLoS One. 2014 Jun 9;9(6):e98756. doi: 10.1371/journal.pone.0098756 (PMC4049617; doi:10.1371/journal.pone.0098756)
Supplement: Table S4 — Fixed parameter values in the model. (DOCX) [file pone.0098756.s011.docx]

**Table S4:** Fixed parameter values in the model

| **Parameter Symbol** | **Parameter Description** | **Value** | **Units** |
| --- | --- | --- | --- |
| Ccadp | Cytosolic adenine diphosphate nucleotide concentration | 0.54 | mM |
| Ccatp | Cytosolic adenine triphosphate nucleotide concentration | 0.31 | mM |
| Ccamp | Cytosolic adenine monophosphate nucleotide concentration | 0.03 | mM |
| pHm | Mitochondrial pH | 8 |  |
| pHi | Intracellular pH | 7.3 |  |
| Mg | Cytosolic magnesium concentration | 0.7 | mM |
| MgADP | Cytosolic MgADP concentration | 0.46 | mM |
| MgATP | Cytosolic MgATP concentration | 2.69 | mM |
| Ncd | Total cytosolic NAD concentration | 0.3 | mM |
| Ccpi | Cytosolic phosphate concentration | 2.5 | mM |
| Cmpyr | Mitochondrial pyruvate concentration | 0.1 | mM |
| Cclac | Cytosolic lactate concentration | 2.5 | mM |
| Cc23p2g | Cytosolic 2,3-bisphosphoglycerate | 3 | mM |
| Ccnad | Cytosolic NAD concentration | 0.299 | mM |
| Ccnadh | Cytosolic NADH concentration | 0.001 | mM |
| Ccala | Cytosolic alanine concentration | 0.2 | mM |
| Ccgsh | Cytosolic glutathione (reduced) concentration | 0.57 | mM |
