# Supplementary material for: Bistability in Glycolysis Pathway as a Physiological Switch in Energy Metabolism
Source: PLoS One. 2014 Jun 9;9(6):e98756. doi: 10.1371/journal.pone.0098756 (PMC4049617; doi:10.1371/journal.pone.0098756)
Supplement: Information S1 — Supplementary information. (DOCX) [file pone.0098756.s012.docx]

**Supplementary Information**

[Rate Equations 1](#_Toc387654688)

[Glycolysis Enzyme Rate Equations 1](#_Toc387654689)

[Description of Transport 9](#_Toc387654690)

[Mathematical Model of Glycolysis Fluxes 10](#_Toc387654691)

[Stability Analysis 11](#_Toc387654692)

[References 12](#_Toc387654693)

# Rate Equations

Kinetic rate equations for all the enzymes used in the model have all been previously derived mechanistically and reported in various literature. This section describes these kinetic equations for all the enzymes considered. The steady state kinetics for all the enzymes were based on the King and Altman method [[1](#_ENREF_1)] employing the known mechanisms and regulations. The rate equations for the enzymes phosphofructokinase (PFK) and pyruvate kinase (PK) employ the Monod-Wyman-Changeux method [[2](#_ENREF_2)] to model the allosteric effects of various metabolite modulators. Kinetic constants used in the rate equations of the current model were experimentally determined values and obtained from previously reported studies.

### Glycolysis Enzyme Rate Equations

**Hexokinase (HK)**: The rate equation for HK was taken from Mulquiney et al. [[3](#_ENREF_3)]. The kinetic constants which correspond to those of the isozyme HK2 were adopted from previous literature [[4-8](#_ENREF_4)]. The rate equation employs the partial rapid equilibrium random bi bi mechanism, which is a simplification of the steady state random bi bi system with the assumption that except for the reactive-ternary complexes, all the other steps in the mechanism are fast reactions. The inhibitions by G6P, glucose-1,6-phosphate (G16BP), 2,3-bisphosphoglycerate (23BPG) and glutathione (GSH) were modeled as mixed type of inhibition affecting both the activity (V_max_) as well as the affinity (K_M_) of the enzyme for glucose.

$$\begin{matrix} r_{HK}=\left(V_{mf}^{HK}\frac{C_{MgATP}^{c}C_{GLC}^{c}}{K_{MgATP}^{HK}K_{GLC}^{HK}}-V_{mr}^{HK}\frac{C_{MgADP}^{c}C_{G6P}^{c}}{K_{i,MgADP}^{HK}K_{G6P}^{HK}}\right)\frac{1}{N_{HK}} \\ N_{HK}=\left(1+\frac{C_{MgATP}^{c}}{K_{i,MgATP}^{HK}}+\frac{C_{G6P}^{c}}{K_{i,G6P}^{HK}}+\frac{C_{GLC}^{c}}{K_{GLC}^{HK}}+\frac{C_{MgATP}^{c}C_{GLC}^{c}}{K_{MgATP}^{HK}K_{GLC}^{HK}}+\frac{C_{MgADP}^{c}}{K_{i,MgADP}^{HK}}+\frac{C_{MgADP}^{c}C_{G6P}^{c}}{K_{i,MgADP}^{HK}K_{G6P}^{HK}}\right.\text{ } \\ \text{ }\left.+\frac{C_{GLC}^{c}C_{G6P}^{c}}{K_{GLC}^{HK}K_{G6P}^{HK}}+\frac{C_{GLC}^{c}C_{G16BP}^{c}}{K_{GLC}^{HK}K_{i,G16BP}^{HK}}+\frac{C_{GLC}^{c}C_{23BPG}^{c}}{K_{GLC}^{HK}K_{i,23BPG}^{HK}}+\frac{C_{GLC}^{c}C_{GSH}^{c}}{K_{GLC}^{HK}K_{i,GSH}^{HK}}\right) \end{matrix}$$

$$\begin{matrix} V_{mf}^{HK}=6.38*10^{3}mM\text{ }h^{-1} \\ V_{mr}^{HK}=41\text{ }mM\text{ }h^{-1} \\ K_{MgATP}^{HK}=1.0\text{ }mM \\ K_{GLC}^{HK}=0.1\text{ }mM \\ K_{i,MgADP}^{HK}=1.0\text{ }mM \\ K_{G6P}^{HK}=0.47\text{ }mM \\ K_{i,G6P}^{"HK}=0.47\text{ }mM \\ K_{i,G16BP}^{HK}=0.03\text{ }mM \\ K_{i,GSH}^{HK}=3.0\text{ }mM \\ K_{i,23BPG}^{HK}=4.0\text{ }mM \end{matrix}$$

**Glucose Phosphate Isomerase (GPI)**: The rate equation for GPI was taken from Mulquiney et al. [[3](#_ENREF_3)]. The kinetic constants were adopted from previous literature [[9-11](#_ENREF_9)]. The rate equation employs the steady state uni uni reaction kinetics.

$$r_{GPI}=\frac{V_{mf}^{GPI}\frac{C_{G6P}^{c}}{K_{f}^{GPI}}-V_{mr}^{GPI}\frac{C_{F6P}^{c}}{K_{r}^{GPI}}}{1+\frac{C_{G6P}^{c}}{K_{f}^{GPI}}+\frac{C_{F6P}^{c}}{K_{r}^{GPI}}}\text{ }$$

$$\begin{matrix} V_{mf}^{GPI}=4.8*10^{4}mM\text{ }h^{-1} \\ V_{mr}^{GPI}=\text{ }4.0*10^{4}mM\text{ }h^{-1} \\ K_{f}^{GPI}=0.3\text{ }mM \\ K_{r}^{GPI}=0.123\text{ }mM \end{matrix}$$

**Phosphofructokinase (PFK)**: The rate equation for PFK was taken from Mulquiney et al. [[3](#_ENREF_3)]. The kinetic constants were adopted from previous literature [[12-16](#_ENREF_12)]. The rate kinetics was based on the two state allosteric model using ordered bi bi mechanism. The two state model considers that the enzyme can exist in the active or the non-active state as determined by the levels of the activity modulators. These include activators (F6P, F16BP, F26BP, G16BP*,* AMP etc) and inhibitors (ATP, Mg etc). Some of these activity modulators act on all the isozymes of PFK while others are isozyme specific. For example, F6P acts as a substrate as well as an allosteric activator for all the isozymes of PFK, whereas F16BP only stimulates PFKM and PFKL. The fraction of enzyme in the active state is represented by the nonlinear term *N_PFK_* which is a function of the levels of the activity modulators. *L_PFK_* represents the equilibrium constant between the two states of the enzyme in the absence of any substrates. The initial velocity expression for the enzyme fraction in the active state was modeled as partial rapid equilibrium random bi bi steady state equation similar to the HK kinetics.

$$\begin{matrix} r_{PFK}=\frac{\frac{V_{f}^{PFK}C_{MgATP}^{c}C_{F6P}^{c}}{K_{F6P}^{PFK}K_{MgATP}^{PFK}}-\frac{V_{r}^{PFK}C_{MgADP}^{c}C_{F16BP}^{c}}{K_{F16BP}^{PFK}K_{MgADP}^{PFK}}}{\left(\left(1+\frac{C_{F6P}^{c}}{K_{F6P}^{PFK}}\right)\left(1+\frac{C_{MgATP}^{c}}{K_{MgATP}^{PFK}}\right)+\left(1+\frac{C_{F16BP}^{c}}{K_{F16BP}^{PFK}}\right)\left(1+\frac{C_{MgADP}^{c}}{K_{MgADP}^{PFK}}\right)-1\right)}\frac{1}{N_{PFK}} \\ N_{PFK}=1+\frac{L_{PFK}\left(1+\frac{C_{ATP}^{c}}{K_{ATP}^{PFK}}\right)^{4}\left(1+\frac{C_{Mg}^{c}}{K_{Mg}^{PFK}}\right)^{4}\left(1+\frac{C_{2,3BPG}^{c}}{K_{2,3BPG}^{PFK}}\right)^{4}}{\left(1+\frac{C_{F6P}^{c}}{K_{F6P}^{PFK}}+\frac{C_{F16BP}^{c}}{K_{F16BP}^{PFK}}\right)^{4}\left(1+\frac{C_{AMP}^{c}}{K_{AMP}^{PFK}}\right)^{4}\left(1+\frac{C_{G16BP}^{c}}{K_{G16BP}^{PFK}}\right)^{4}\left(1+\frac{C_{Pi}^{c}}{K_{Pi}^{PFK}}\right)^{4}\left(1+\frac{C_{F26BP}^{c}}{K_{F26BP}^{PFK}}\right)^{4}} \\ \\ \\ \\ \\ \\ \\ \\ \end{matrix}$$

$$\begin{matrix} V_{f}^{PFK}=15.5*10^{2}mM^{-1}h^{-1} \\ V_{r}^{PFK}=6.78*10^{1}mM^{-1}h^{-1} \\ K_{F6P}^{PFK}=6*10^{-2}\text{ }mM \\ K_{MgATP}^{PFK}=6.8*10^{-2}\text{ }mM \\ K_{MgADP}^{PFK}=0.54\text{ }mM \\ K_{F16BP}^{PFK}=0.65\text{ }mM \\ K_{F26BP}^{PFK}=5.5*10^{-3}mM \\ K_{G16BP}^{PFK}=0.1\text{ }mM \\ K_{ATP}^{PFK}=0.1\text{ }mM \\ K_{AMP}^{PFK}=0.3\text{ }mM \\ K_{Mg}^{PFK}=0.2\text{ }mM \\ K_{Pi}^{PFK}=30\text{ }mM \\ K_{23BPG}^{PFK}=0.5\text{ }mM \\ L_{PFK}=2*10^{-3} \end{matrix}$$

**6-Phosphofructo-2-Kinase/Fructose-2,6-Bisphosphatase (PFKFB)**: The rate equation for PFKFB and the kinetic constants were taken from previously reported studies [[17](#_ENREF_17),[18](#_ENREF_18)]. PFKFB is a bi-functional enzyme with kinase and bisphosphatase activities, each localized to either terminals of the enzyme and are independent of each other’s activity. The kinase domain catalyzes the synthesis of fructose-2,6-bisphosphate (F26BP) from fructose-6-phoshate (F6P) and the bisphosphatase domain mediates the hydrolysis of F26BP to F6P. The reaction kinetics for the kinase domain (r_PFK2_) follows the ordered bi bi steady state kinetics, with phosphoenolpyurvate (PEP) inhibition of the kinase domain modeled as non-competitive inhibition. The bisphosphatase reaction kinetics (r_F2,6BPase_) was modeled as simple Michaelis-Menten kinetics with non-competitive product inhibition by F6P. Isozymes of PFKFB vary in their kinase to bisphosphatase activity (K/P) [[19](#_ENREF_19)]. The effect of isozyme (or K/P) was modeled by changing the Vmax of r_PFK2_ and holding r_F2,6BPase_ constant.

$\begin{matrix} r_{PFK2}=\frac{V_{f,PFK2}\left(C_{ATP}^{c}C_{F6P}^{c}-\frac{C_{ADP}^{c}C_{F26BP}^{c}}{K_{eq,PFK2}}\right)}{\left(K_{i,ATP}^{PFK2}K_{m,F6P}^{PFK2}+K_{m,F6P}^{PFK2}C_{ATP}^{c}+K_{m,ATP}^{PFK2}C_{F6P}^{c}+\frac{K_{m,ADP}^{PFK2}C_{F26B[}^{c}}{K_{eq,PFK2}}+\frac{K_{m,F26B[}^{PFK2}C_{ADP}^{c}}{K_{eq,PFK2}}\right.} \\ \begin{matrix} & & + \end{matrix}C_{ATP}^{c}C_{F6P}^{c}+\frac{K_{m,ADP}^{PFK2}C_{ATP}^{c}C_{F26BP}^{c}}{K_{eq,PFK2}K_{i,ATP}^{PFK2}}+\frac{C_{ADP}^{c}C_{F26BP}^{c}}{K_{eq,PFK2}}+\frac{K_{m,ATP}^{PFK2}C_{ADP}^{c}C_{F6P}^{c}}{K_{i,ADP}^{PFK2}} \\ \begin{matrix} & & + \end{matrix}\left.\frac{C_{ATP}^{c}C_{F6P}^{c}C_{F26BP}^{c}}{K_{i,F26BP}^{PFK2}}+\frac{C_{ADP}^{c}C_{F6P}^{c}C_{F26BP}^{c}}{K_{eq,PFK2}K_{i,F6P}^{PFK2}}\right)\left(1+\frac{C_{PEP}^{c}}{K_{i,PEP}^{PFK2}}\right) \end{matrix}$

$$\begin{matrix} V_{f,PFK2}=41.6\text{ }mM\text{ }h^{-1} \\ K_{m,ATP}^{PFK2}=0.15\text{ }mM \\ K_{m,F6P}^{PFK2}=0.032\text{ }mM \\ K_{m,F26BP}^{PFK2}=0.008\text{ }mM \\ K_{m,ADP}^{PFK2}=0.062\text{ }mM \\ K_{i,ATP}^{PFK2}=0.15\text{ }mM \\ K_{i,F6P}^{PFK2}=0.001\text{ }mM \\ K_{i,F26BP}^{PFK2}=0.02\text{ }mM \\ K_{i,ADP}^{PFK2}=0.23\text{ }mM \\ K_{i,PEP}^{PFK2}=0.013\text{ }mM \\ K_{eq}^{PFK2}=16\text{ } \end{matrix}$$

$$r_{F2,6BPase}=\frac{V_{F2,6BPase}C_{F26BP}^{c}}{\left(1+\frac{C_{F6P}^{c}}{K_{i,F6P}^{F2,6BPase}}\right)\left(K_{m,F26BP}^{F2,6BPase}+C_{F26BP}^{c}\right)}$$

$$\begin{matrix} V_{F2,6BPase}=11.78\text{ }mM^{-1}h^{-1} \\ K_{m,F26BP}^{F2,6BPase}=1*10^{-3}\text{ }mM \\ K_{i,F6P}^{F2,6BPase}=25*10^{-3}\text{ }mM \end{matrix}$$

**Aldolase (ALDO):** The rate equation for ALDO was taken from Mulquiney et al. [[3](#_ENREF_3)]. The kinetic constants were adopted or estimated from previous literature [[20-29](#_ENREF_20)]. The reaction kinetics of ALDO follows the ordered uni bi steady state kinetics. Inhibition due to 23BPG as described in the original expression was retained in this study. However, since 23BPG is not a reaction intermediate considered in the model, its concentration was held constant for the purpose of this study.

$$\begin{matrix} r_{ALD}=\frac{\frac{V_{mf}^{ALD}C_{F16BP}^{c}}{K_{F16BP}^{ALD}}-\frac{V_{mr}^{ALD}C_{GAP}^{c}C_{DHAP}^{c}}{K_{GAP}^{ALD}K_{i,DHAP}^{ALD}}}{\left(1+\frac{C_{23BPG}^{c}}{K_{i,23BPG}^{ALD}}+\frac{C_{F16BP}^{c}}{K_{F16BP}^{ALD}}+\frac{K_{DHAP}^{ALD}C_{GAP}^{c}}{K_{GAP}^{ALD}K_{i,DHAP}^{ALD}}\left(1+\frac{C_{23BPG}^{c}}{K_{i,23BPG}^{ALD}}\right)+\frac{C_{DHAP}^{c}}{K_{i,DHAP}^{ALD}}\right.} \\ \begin{matrix} \begin{matrix} & \end{matrix} & \end{matrix}\left.+\frac{K_{DHAP}^{ALD}C_{F16BP}^{c}C_{GAP}^{c}}{K_{i,F16BP}^{ALD}K_{GAP}^{ALD}K_{i,DHAP}^{ALD}}+\frac{C_{DHAP}^{c}C_{GAP}^{c}}{K_{GAP}^{ALD}K_{i,DHAP}^{ALD}}\right) \end{matrix}$$

$$\begin{matrix} V_{mf}^{ALD}=6.75*10^{2}mM\text{ }h^{-1} \\ V_{mr}^{ALD}=\text{ }2.32*10^{3}mM\text{ }h^{-1} \\ K_{F16BP}^{ALD}=5*10^{-2}mM \\ K_{i,F16BP}^{ALD}=1.98*10^{-2}mM \\ K_{DHAP}^{ALD}=3.5*10^{-2}mM \\ K_{i,DHAP}^{ALD}=1.1*10^{-2}mM \\ K_{GAP}^{ALD}=0.189mM \\ K_{i,23BPG}^{ALD}=1.5mM \end{matrix}$$

**Triose Phosphate Isomerase (TPI)**: The rate equation for TPI was taken from Mulquiney et al. [[3](#_ENREF_3)]. The kinetic constants were adopted from previous literature [[29-33](#_ENREF_29)]. The rate kinetics of TPI follows a simple steady state uni uni reaction kinetics.

$$r_{TPI}=\frac{V_{mf}^{TPI}\frac{C_{DHAP}^{c}}{K_{f}^{TPI}}-V_{mr}^{TPI}\frac{C_{GAP}^{c}}{K_{r}^{TPI}}}{1+\frac{C_{DHAP}^{c}}{K_{f}^{TPI}}+\frac{C_{GAP}^{c}}{K_{r}^{TPI}}}$$

$$\begin{matrix} V_{mf}^{TPI}=5.10*10^{2}mM\text{ }h^{-1} \\ V_{mr}^{TPI}=4.61*10^{1}mM\text{ }h^{-1} \\ K_{f}^{TPI}=1.62*10^{-1}mM \\ K_{r}^{TPI}=4.30*10^{-1}mM \end{matrix}$$

**Glyceraldehyde 3-Phosphate Dehydrogenase (GAPDH)**: The rate equation for GAPDH was taken from Mulquiney et al. [[3](#_ENREF_3)]. The kinetic constants were adopted from previous literature [[34-37](#_ENREF_34)]. The rate kinetics of GAPDH follows the ter ter (bi uni uni bi ping pong) steady state kinetics.

$$\begin{matrix} r_{GAPD}=\frac{V_{mf}^{GAPD}\frac{C_{NAD^{+}}^{c}C_{Pi}^{c}C_{GAP}^{c}}{K_{NAD^{+}}^{GAPD}K_{i,Pi}^{GAPD}K_{i,GAP}^{GAPD}}-V_{mr}^{GAPD}\frac{C_{13BPG}^{c}C_{NADH}^{c}C_{H^{+}}^{c}}{K_{i,13BPG}^{GAPD}K_{NADH}^{GAPD}}}{\begin{matrix} \frac{C_{GAP}^{c}}{K_{i,GAP}^{GAPD}}\left(1+\frac{C_{GAP}^{c}}{K_{i,GAP}^{'GAPD}}\right)+\frac{C_{13BPG}^{c}}{K_{i,13BPG}^{GAPD}}\left(1+\frac{C_{GAP}^{c}}{K_{i,GAP}^{'GAPD}}\right)+\frac{K_{13BPG}^{GAPD}C_{NADH}^{c}C_{H^{+}}^{c}}{K_{i,13BPG}^{GAPD}K_{NADH}^{GAPD}}+\frac{K_{GAP}^{GAPD}C_{NAD^{+}}^{c}C_{Pi}^{c}}{K_{NAD^{+}}^{GAPD}K_{i,Pi}^{GAPD}K_{i,GAP}^{GAPD}} \\ +\frac{C_{NAD^{+}}^{c}C_{GAP}^{c}}{K_{i,NAD^{+}}^{GAPD}K_{i,GAP}^{GAPD}}+\frac{C_{Pi}^{c}C_{GAP}^{c}}{K_{i,Pi}^{GAPD}K_{i,GAP}^{GAPD}}\left(1+\frac{C_{GAP}^{c}}{K_{i,GAP}^{'GAPD}}\right)+\frac{C_{NAD^{+}}^{c}C_{13BPG}^{c}}{K_{i,NAD^{+}}^{GAPD}K_{i,13BPG}^{GAPD}}+\frac{K_{13BPG}^{GAPD}C_{Pi}^{c}C_{NADH}^{c}C_{H^{+}}^{c}}{K_{i,Pi}^{GAPD}K_{i,13BPG}^{GAPD}K_{NADH}^{GAPD}} \\ +\frac{C_{GAP}^{c}C_{NADH}^{c}C_{H^{+}}^{c}}{K_{i,GAP}^{GAPD}K_{i,NADH}^{GAPD}}+\frac{C_{13BPG}^{c}C_{NADH}^{c}C_{H^{+}}^{c}}{K_{i,13BPG}^{GAPD}K_{NADH}^{GAPD}}+\frac{C_{NAD^{+}}^{c}C_{Pi}^{c}C_{GAP}^{c}}{K_{NAD^{+}}^{GAPD}K_{i,Pi}^{GAPD}K_{i,GAP}^{GAPD}}+\frac{K_{GAP}^{GAPD}C_{NAD^{+}}^{c}C_{Pi}^{c}C_{13BPG}^{c}}{K_{i,GAP}^{GAPD}K_{NAD^{+}}^{GAPD}K_{i,Pi}^{GAPD}K_{i,13BPG}^{'GAPD}} \\ +\frac{C_{Pi}^{c}C_{GAP}^{c}C_{NADH}^{c}C_{H^{+}}^{c}}{K_{i,Pi}^{GAPD}K_{i,GAP}^{GAPD}K_{i,NADH}^{GAPD}}+\frac{C_{Pi}^{c}C_{13BPG}^{c}C_{NADH}^{c}C_{H^{+}}^{c}}{K_{i,13BPG}^{GAPD}K_{NADH}^{GAPD}K_{i,Pi}^{GAPD}K_{i,13BPG}^{'GAPD}} \end{matrix}} \\ \\ \end{matrix}$$

$$\begin{matrix} V_{mf}^{GAPD}=5.317*10^{3}mMh^{-1} \\ V_{mr}^{GAPD}=3.919*10^{3}mMh^{-1} \\ K_{NAD^{+}}^{GAPD}=0.045mM \\ K_{i,NAD^{+}}^{GAPD}=0.045mM \\ K_{Pi}^{GAPD}=3.16mM \\ K_{i,Pi}^{GAPD}=3.16mM \\ K_{GAP}^{GAPD}=0.095mM \\ K_{i,GAP}^{GAPD}=1.59*10^{-16}mM \\ K_{i,GAP}^{'GAPD}=0.031mM \\ K_{NADH}^{GAPD}=0.0033mM \\ K_{i,NADH}^{GAPD}=0.01mM \\ K_{13BPG}^{GAPD}=0.00671mM \\ K_{i,13BPG}^{GAPD}=1.52*10^{-18}mM \\ K_{i,13BPG}^{'GAPD}=0.001mM \\ K_{eq}^{GAPD}=1.9*10^{-8} \end{matrix}$$

**Phosphoglycerate Kinase (PGK)**: The rate equation for PGK was taken from Mulquiney et al. [[3](#_ENREF_3)]. The kinetic constants were adopted from previous literature [[38-41](#_ENREF_38)]. The rate kinetics of PGK follows the partial rapid equilibrium random bi bi steady state kinetics.

$$r_{PGK}=\frac{V_{mf}^{PGK}\frac{C_{13BPG}^{c}C_{MgADP}^{c}}{K_{i,MgADP}^{PGK}K_{13BPG}^{PGK}}-V_{mr}^{PGK}\frac{C_{3PG}^{c}C_{MgATP}^{c}}{K_{i,MgATP}^{PGK}K_{3PG}^{PGK}}}{1+\frac{C_{13BPG}^{c}}{K_{i,13BPG}^{PGK}}+\frac{C_{MgADP}^{c}}{K_{i,MgADP}^{PGK}}+\frac{C_{13BPG}^{c}C_{MgADP}^{c}}{K_{i,MgADP}^{PGK}K_{13BPG}^{PGK}}+\frac{C_{3PG}^{c}}{K_{i,3PG}^{PGK}}+\frac{C_{MgATP}^{c}}{K_{i,MgATP}^{PGK}}+\frac{C_{3PG}^{c}C_{MgATP}^{c}}{K_{i,MgATP}^{PGK}K_{3PG}^{PGK}}}$$

$$\begin{matrix} V_{mf}^{PGK}=\text{5.96*10}^{4}mMh^{-1} \\ V_{mr}^{PGK}=2.39\text{*10}^{4}mMh^{-1} \\ K_{MgADP}^{PGK}=0.1mM \\ K_{i,MgADP}^{PGK}=0.08mM \\ K_{13BPG}^{PGK}=0.002mM \\ K_{i,13BPG}^{PGK}=1.6mM \\ K_{MgATP}^{PGK}=1mM \\ K_{i,MgATP}^{PGK}=0.186mM \\ K_{3PG}^{PGK}=1.1mM \\ K_{i,3PG}^{PGK}=0.205mM \\ K_{eq}^{PGK}=3.2*10^{3} \end{matrix}$$

**Phosphoglycerate Mutase (PGM)**: The rate equation for PGM was taken from Mulquiney et al. [[3](#_ENREF_3)]. The kinetic constants were adopted from previous literature [[42](#_ENREF_42),[43](#_ENREF_43)]. The rate kinetics of PGM follows the uni uni steady state kinetics.

$$r_{PGAM}=\frac{V_{mf}^{PGAM}\frac{C_{3PG}^{c}}{K_{3PG}^{PGAM}}-V_{mr}^{PGAM}\frac{C_{2PG}^{c}}{K_{2PG}^{PGAM}}}{1+\frac{C_{3PG}^{c}}{K_{3PG}^{PGAM}}+\frac{C_{2PG}^{c}}{K_{2PG}^{PGAM}}}$$

$$\begin{matrix} V_{mf}^{PGAM}=\text{4.894*10}^{5}mMh^{-1} \\ V_{mr}^{PGAM}=\text{4.395*10}^{5}mMh^{-1} \\ K_{3PG}^{PGAM}=0.168mM \\ K_{2PG}^{PGAM}=0.0256mM \\ K_{eq}^{PGAM}=0.17 \end{matrix}$$

**Enolase (ENO)**: The rate equation for ENO was taken from Mulquiney et al. [[3](#_ENREF_3)]. The kinetic constants were adopted from previous literature [[44-46](#_ENREF_44)]. The rate kinetics of ENO follows the partial rapid equilibrium random bi bi steady state kinetics.

$$r_{ENO}=\frac{V_{mf}^{ENO}\frac{C_{2PG}^{c}C_{Mg}^{c}}{K_{i,Mg}^{ENO}K_{2PG}^{ENO}}-V_{mr}^{ENO}\frac{C_{PEP}^{c}C_{Mg}^{c}}{K_{i,Mg}^{ENO}K_{PEP}^{ENO}}}{1+\frac{C_{2PG}^{c}}{K_{i,2PG}^{ENO}}+\frac{C_{Mg}^{c}}{K_{i,Mg}^{ENO}}+\frac{C_{2PG}^{c}C_{Mg}^{c}}{K_{i,Mg}^{ENO}K_{2PG}^{ENO}}+\frac{C_{PEP}^{c}}{K_{i,PEP}^{ENO}}+\frac{C_{Mg}^{c}}{K_{i,Mg}^{ENO}}+\frac{C_{PEP}^{c}C_{Mg}^{c}}{K_{i,Mg}^{ENO}K_{PEP}^{ENO}}}$$

$$\begin{matrix} V_{mf}^{ENO}=\text{ 2.106*10}^{4}mMh^{-1} \\ V_{mr}^{ENO}=\text{5.542*10}^{3}mMh^{-1} \\ K_{i,Mg}^{ENO}=K_{Mg}^{ENO}=0.14mM \\ K_{PEP}^{ENO}=K_{PEP}^{ENO}=0.11mM \\ K_{2PG}^{ENO}=K_{2PG}^{ENO}=0.046mM \\ K_{eq}^{ENO}=3.0 \end{matrix}$$

**Pyruvate Kinase (PK)**: The rate equation for PK was taken from Mulquiney et al. [[3](#_ENREF_3)]. The kinetic constants were adopted from previous literature [[47-52](#_ENREF_47)]. Like PFK, the rate kinetics of PK was based on the two state allosteric model using the ordered bi bi mechanism. The two state model considers that the enzyme can exist in active or non-active state determined by the levels of the activity modulators. These include activators (F16BP, PEP, PYR etc) and inhibitors (ATP*,* ALA etc). The fraction of the enzyme in the active state is represented by the nonlinear term *N_PK_* which is a function of levels of activity modulators. *L_PK_* represents the equilibrium constant between enzymes at the two states in the absence of any substrates. The initial velocity expression for the enzyme fraction in the active state is modeled as partial rapid equilibrium random bi bi steady state equation.

$$\begin{matrix} r_{PK}=\left(\frac{V_{mf}^{PK}\frac{C_{PEP}^{c}}{K_{PEP}^{PK}}\frac{C_{MgADP}^{c}}{K_{MgADP}^{PK}}-V_{mr}^{PK}\frac{C_{PYR}^{c}}{K_{PYR}^{PK}}\frac{C_{MgATP}^{c}}{K_{MgATP}^{PK}}}{\left(1+\frac{C_{PEP}^{c}}{K_{PEP}^{PK}}\right)\left(1+\frac{C_{MgADP}^{c}}{K_{MgADP}^{PK}}\right)+\left(1+\frac{C_{PYR}^{c}}{K_{PYR}^{PK}}\right)\left(1+\frac{C_{MgATP}^{c}}{K_{MgATP}^{PK}}\right)-1}\right)\frac{1}{N_{PK}}\text{ } \\ \text{ } \\ N_{PK}=1+L_{PK}\frac{\left(1+\frac{C_{ATP}^{c}}{K_{ATP}^{PK}}\right)^{4}\left(1+\frac{C_{ALA}^{c}}{K_{ALA}^{PK}}\right)^{4}}{\left(1+\frac{C_{PEP}^{c}}{K_{PEP}^{PK}}+\frac{C_{PYR}^{c}}{K_{PYR}^{PK}}\right)^{4}\left(1+\frac{C_{F16BP}^{c}}{K_{F16BP}^{PK}}+\frac{C_{G16BP}^{c}}{K_{G16BP}^{PK}}\right)^{4}} \end{matrix}$$

$$\begin{matrix} V_{mf}^{PK}=2.02*10^{4}mM\text{ }h^{-1}\text{ } \\ V_{mf}^{PK}=47.5mM\text{ }h^{-1} \\ K_{PEP}^{PK}=2.25*10^{-1}mM \\ K_{MgADP}^{PK}=4.74*10^{-1}mM \\ K_{MgATP}^{PK}=3\text{ }mM \\ K_{ATP}^{PK}=3.39\text{ }mM \\ K_{PYR}^{PK}=4\text{ }mM \\ K_{F16BP}^{PK}=0.04\text{ }mM \\ K_{G16BP}^{PK}=1.0*10^{-1}mM \\ L_{PK}=0.398 \\ K_{ALA}^{PK}=0.02\text{ }mM \end{matrix}$$

**Lactate Dehydrogenase (LDH)**: The rate equation for LDH and the kinetic constants were adopted from previous literature [[3](#_ENREF_3),[53-55](#_ENREF_53)]. The kinetics of LDH was modeled as ordered bi bi steady state kinetics, with substrate inhibition by pyruvate.

$$r_{LDH}=\frac{V_{mf}^{LDH}\frac{C_{NADH}^{c}C_{PYR}^{c}}{K_{i,NADH}^{LDH}K_{PYR}^{LDH}}-V_{mr}^{LDH}\frac{C_{NAD}^{c}C_{LAC}^{c}}{K_{i,NAD}^{LDH}K_{LAC}^{LDH}}}{\begin{matrix} \left(1+\frac{K_{NADH}^{LDH}C_{PYR}^{c}}{K_{i,NADH}^{LDH}K_{PYR}^{LDH}}+\frac{K_{NAD}^{LDH}C_{LAC}^{c}}{K_{i,NAD}^{LDH}K_{LAC}^{LDH}}\right)\left(1+\frac{C_{PYR}^{c}}{K_{i,PYR}^{"LDH}}\right)+\frac{C_{NADH}^{c}}{K_{i,NADH}^{LDH}}+\frac{C_{NAD}^{c}}{K_{i,NAD}^{LDH}} \\ +\frac{C_{NADH}^{c}C_{PYR}^{c}}{K_{i,NADH}^{LDH}K_{PYR}^{LDH}}+\frac{K_{NAD}^{LDH}C_{NADH}^{c}C_{LAC}^{c}}{K_{i,NAD}^{LDH}K_{i,NADH}^{LDH}K_{LAC}^{LDH}}+\frac{K_{NADH}^{LDH}C_{NAD}^{c}C_{PYR}^{c}}{K_{i,NAD}^{LDH}K_{i,NADH}^{LDH}K_{PYR}^{LDH}}+\frac{C_{NAD}^{c}C_{LAC}^{c}}{K_{i,NAD}^{LDH}K_{LAC}^{LDH}} \\ +\frac{C_{NADH}^{c}C_{PYR}^{c}C_{LAC}^{c}}{K_{i,NADH}^{LDH}K_{PYR}^{LDH}K_{i,LAC}^{LDH}}+\frac{C_{NAD}^{c}C_{PYR}^{c}C_{LAC}^{c}}{K_{i,NAD}^{LDH}K_{i,PYR}^{LDH}K_{LAC}^{LDH}} \end{matrix}}$$

$$\begin{matrix} V_{mf}^{LDH}=8.66*10^{3}mMh^{-1} \\ V_{mr}^{LDH}=2.17*10^{3}mMh^{-1} \\ K_{PYR}^{LDH}=0.137\text{ }mM \\ K_{i,PYR}^{LDH}=0.228\text{ }mM \\ K_{NAD}^{LDH}=0.107\text{ }mM \\ K_{i,NAD}^{LDH}=0.503\text{ }mM \\ K_{LAC}^{LDH}=1.07\text{ }mM \\ K_{i,LAC}^{LDH}=7.33\text{ }mM \\ K_{i,NADH}^{LDH}=5.45*10^{-3}\text{ }mM \\ K_{NADH}^{LDH}=7.43*10^{-3}\text{ }mM \\ K_{i,PYR}^{"LDH}=0.101\text{ }mM \end{matrix}$$

### Description of Transport

**Glucose Transporter (GLUT)**: Glucose transporters mediate transport of glucose across plasma membranes. Till date, fourteen glucose transporters (isozymes) have been identified which perform the same function but have very different kinetic properties [[56](#_ENREF_56)]. Kinetics of the GLUT1 isozyme was considered in the model and was modeled as uni uni steady state kinetics.

$$r_{GLUT}=\frac{V_{mf}^{GLUT}\frac{C_{GLC}^{e}}{K_{GLC}^{GLUT}}-V_{mr}^{GLUT}\frac{C_{GLC}^{c}}{K_{GLC}^{GLUT}}}{1+\frac{C_{GLC}^{e}}{K_{GLC}^{GLUT}}+\frac{C_{GLC}^{c}}{K_{GLC}^{GLUT}}}$$

$$\begin{matrix} V_{mf}^{GLUT}=7.67\text{ mM}h^{-1} \\ V_{mr}^{GLUT}=0.767\text{ mM}h^{-1} \\ K_{GLC}^{GLUT}=1.50\text{ }mM \end{matrix}$$

**Mitochondrial Pyruvate Transporter**: Rate equation for pyruvate transport into mitochondrion was modeled as reversible first ordered mass kinetics.

$$r_{PYRH}=V_{mf}^{PYRH}\left(C_{PYR}^{c}C_{H^{+}}^{c}-C_{PYR}^{m}C_{H^{+}}^{m}\right)$$

$$V_{mf}^{PYRH}\text{=6.67*10}^{12}\text{ }mM^{-1}\text{ }h^{-1}$$

# Mathematical Model of Glycolysis Fluxes

The mathematical model for the cellular metabolism consists of material balance equations for each reaction intermediates in glycolysis. The reaction equations are from the mechanistic equations shown in the previous section. The dilution effect on metabolite concentrations caused by the cell growth was neglected considering the difference of at least one order of magnitude between the time constant for growth and specific glucose consumption rate.

1. Glucose:$\frac{dC_{GLC}^{c}}{dt}=r_{GLUT}-r_{HK}$
2. Glucose 6-phosphate: $\frac{dC_{G6P}^{c}}{dt}=r_{HK}-r_{GPI}$
3. Fructose 6-phosphate: $\frac{dC_{F6P}^{c}}{dt}=r_{GPI}-r_{PFK}-r_{PFK2}+r_{F2,6BPase}$
4. Fructose 1,6-bisphosphate: $\frac{dC_{F16BP}^{c}}{dt}=r_{PFK}-r_{ALD}$
5. Fructose 2,6-bisphosphate: $\frac{dC_{F26BP}^{c}}{dt}=r_{PFK2}-r_{F2,6BPase}$
6. Dihydroxyacetone phosphate: $\frac{dC_{DHAP}^{c}}{dt}=r_{ALD}-r_{TPI}$
7. Glyceraldehyde 3-phosphate: $\frac{dC_{GAP}^{c}}{dt}=r_{ALD}+r_{TPI}-r_{GAPD}$
8. 1,3-bisphosphoglycerate: $\frac{dC_{13BPG}^{c}}{dt}=r_{GAPD}-r_{PGK}$
9. 3-phosphoglycerate: $\frac{dC_{3PG}^{c}}{dt}=r_{PGK}-r_{PGM}$
10. 2-phosphoglycerate: $\frac{dC_{2PG}^{c}}{dt}=r_{PGM}-r_{EN}$
11. Phosphoenolpyruvate: $\frac{dC_{PEP}^{c}}{dt}=r_{EN}-r_{PK}$
12. Pyruvate: $\frac{dC_{PYR}^{c}}{dt}=r_{PK}-r_{LDH}-r_{PYRH}$

# Stability Analysis

For a system of ordinary differential equation $\frac{dx}{dt}=f\left(x\right)$, where $x\in R^{n}$and $f:R^{n}\to R^{n}$, the Jacobian matrix, $J$ is the $n\times n$matrix defined by $J_{ij}=\partial f_{i}/\partial x_{j}$. The local stability of a steady state was investigated using the standard approach of calculating the eigenvalues of the Jacobian evaluated at the steady state. If all the eigenvalues have negative real part, the steady state is stable, if not it is unstable. The Jacobian matrix was calculated as part of the output of Matlab’s *fsolve* function. The eigenvalues of the Jacobian matrix were evaluated using Matlab’s *eig* function.

# References

1. Segel IH (1975) Enzyme kinetics : behavior and analysis of rapid equilibrium and steady state enzyme systems. New York: Wiley. xxii, 957 p. p.

2. Monod J, Wyman J, Changeux JP (1965) On the Nature of Allosteric Transitions: a Plausible Model. J Mol Biol 12: 88-118.

3. Mulquiney PJ, Kuchel PW (1999) Model of 2,3-bisphosphoglycerate metabolism in the human erythrocyte based on detailed enzyme kinetic equations: equations and parameter refinement. Biochem J 342 Pt 3: 581-596.

4. Gerber G, Preissler H, Heinrich R, Rapoport SM (1974) Hexokinase of human erythrocytes. Purification, kinetic model and its application to the conditions in the cell. Eur J Biochem 45: 39-52.

5. Rijksen G, Jansen G, Kraaijenhagen RJ, Van der Vlist MJ, Vlug AM, et al. (1981) Separation and characterization of hexokinase I subtypes from human erythrocytes. Biochim Biophys Acta 659: 292-301.

6. Rijksen G, Staal GE (1977) Regulation of human erythrocyte hexokinase. The influence of glycolytic intermediates and inorganic phosphate. Biochim Biophys Acta 485: 75-86.

7. Fornaini G, Magnani M, Fazi A, Accorsi A, Stocchi V, et al. (1985) Regulatory properties of human erythrocyte hexokinase during cell ageing. Arch Biochem Biophys 239: 352-358.

8. Magnani M, Stocchi V, Ninfali P, Dacha M, Fornaini G (1980) Action of oxidized and reduced glutathione on rabbit red blood cell hexokinase. Biochim Biophys Acta 615: 113-120.

9. Gracy RW, Tilley BE (1975) Phosphoglucose isomerase of human erythrocytes and cardiac tissue. Methods Enzymol 41: 392-400.

10. Kahana SE, Lowry OH, Schulz DW, Passonneau JV, Crawford EJ (1960) The kinetics of phosphoglucoisomerase. J Biol Chem 235: 2178-2184.

11. Tilley BE, Gracy RW, Welch SG (1974) A point mutation increasing the stability of human phosphoglucose isomerase. J Biol Chem 249: 4751-4759.

12. Merry S, Britton HG (1985) The mechanism of rabbit muscle phosphofructokinase at pH8. Biochem J 226: 13-28.

13. Dunaway GA, Kasten TP, Sebo T, Trapp R (1988) Analysis of the phosphofructokinase subunits and isoenzymes in human tissues. Biochem J 251: 677-683.

14. Hanson RL, Rudolph FB, Lardy HA (1973) Rabbit muscle phosphofructokinase. The kinetic mechanism of action and the equilibrium constant. J Biol Chem 248: 7852-7859.

15. Otto M, Heinrich R, Jacobasch G, Rapoport S (1977) A mathematical model for the influence of anionic effectors on the phosphofructokinase from rat erythrocytes. Eur J Biochem 74: 413-420.

16. Otto M, Heinrich R, Kuhn B, Jacobasch G (1974) A mathematical model for the influence of fructose 6-phosphate, ATP, potassium, ammonium and magnesium on the phosphofructokinase from rat erythrocytes. Eur J Biochem 49: 169-178.

17. Kitajima S, Sakakibara R, Uyeda K (1984) Kinetic studies of fructose 6-phosphate,2-kinase and fructose 2,6-bisphosphatase. J Biol Chem 259: 6896-6903.

18. Kretschmer M, Schellenberger W, Hofmann E (1985) Quasi-stationary concentrations of fructose-2,6-bisphosphate in the phosphofructokinase-2/fructose-2,6-bisphosphatase cycle. Biochem Biophys Res Commun 131: 899-904.

19. Okar DA, Manzano A, Navarro-Sabate A, Riera L, Bartrons R, et al. (2001) PFK-2/FBPase-2: maker and breaker of the essential biofactor fructose-2,6-bisphosphate. Trends Biochem Sci 26: 30-35.

20. Srivastava SK, Beutler E (1972) The effect of normal red cell constituents on the activities of red cell enzymes. Arch Biochem Biophys 148: 249-255.

21. Beutler E (1971) 2,3-diphosphoglycerate affects enzymes of glucose metabolism in red blood cells. Nat New Biol 232: 20-21.

22. Mehler AH (1963) Kinetic properties of native and carboxy-peptidase-altered rabbit muscle aldolase. J Biol Chem 238: 100-104.

23. Mehler AH, Bloom B (1963) Interaction between rabbit muscle aldolase and dihydroxyacetone phosphate. J Biol Chem 238: 105-107.

24. Penhoet EE, Kochman M, Rutter WJ (1969) Ioslation of fructose diphosphate aldolases A, B, and C. Biochemistry 8: 4391-4395.

25. Penhoet EE, Kochman M, Rutter WJ (1969) Molecular and catalytic properties of aldolase C. Biochemistry 8: 4396-4402.

26. Strapazon E, Steck TL (1977) Interaction of the aldolase and the membrane of human erythrocytes. Biochemistry 16: 2966-2971.

27. Yeltman DR, Harris BG (1977) Purification and characterization of aldolase from human erythrocytes. Biochim Biophys Acta 484: 188-198.

28. Rose IA, O'Connell EL, Mehler AH (1965) Mechanism of the Aldolase Reaction. J Biol Chem 240: 1758-1765.

29. Beutler E (1984) Red Cell Metabolism: A Manual of Biochemical Methods. New York: Grune and Stratton.

30. Sawyer TH, Tilley BE, Gracy RW (1972) Studies on human triosephosphate isomerase. II. Nature of the electrophoretic multiplicity in erythrocytes. J Biol Chem 247: 6499-6505.

31. Schneider AS, Valentine WN, Hattori M, Heins HL, Jr. (1965) Hereditary Hemolytic Anemia with Triosephosphate Isomerase Deficiency. N Engl J Med 272: 229-235.

32. Meyerhof OaJ-K, R. (1943) The equlibria of isomerase and aldolase, and the problem of the phosphorylation of glyceraldehyde phosphate Journal of Biological Chemistry 1943: 71-92.

33. Gracy RW (1975) Triosephosphate isomerase from human erythrocytes. Methods Enzymol 41: 442-447.

34. Wang CS, Alaupovic P (1980) Glyceraldehyde-3-phosphate dehydrogenase from human erythrocyte membranes. Kinetic mechanism and competitive substrate inhibition by glyceraldehyde 3-phosphate. Arch Biochem Biophys 205: 136-145.

35. Heinz F, Freimuller B (1982) Glyceraldehyde-3-phosphate dehydrogenase from human tissues. Methods Enzymol 89 Pt D: 301-305.

36. Furfine CS, Velick SF (1965) The Acyl-Enzyme Intermediate and the Kinetic Mechanism of the Glyceraldehyde 3-Phosphate Dehydrogenase Reaction. J Biol Chem 240: 844-855.

37. Cori CF, Velick SF, Cori GT (1950) The combination of diphosphopyridine nucleotide with glyceraldehyde phosphate dehydrogenase. Biochim Biophys Acta 4: 160-169.

38. Krietsch WK, Bucher T (1970) 3-phosphoglycerate kinase from rabbit sceletal muscle and yeast. Eur J Biochem 17: 568-580.

39. Yoshida A, Watanabe S (1972) Human phosphoglycerate kinase. I. Crystallization and characterization of normal enzyme. J Biol Chem 247: 440-445.

40. Ali M, Brownstone YS (1976) A study of phosphoglycerate kinase in human erythrocytes. II. Kinetic properties. Biochim Biophys Acta 445: 89-103.

41. Lee CS, O'Sullivan WJ (1975) Properties and mechanism of human erythrocyte phosphoglycerate kinase. J Biol Chem 250: 1275-1281.

42. Mulquiney PJ, Bubb WA, Kuchel PW (1999) Model of 2,3-bisphosphoglycerate metabolism in the human erythrocyte based on detailed enzyme kinetic equations: in vivo kinetic characterization of 2,3-bisphosphoglycerate synthase/phosphatase using 13C and 31P NMR. Biochem J 342 Pt 3: 567-580.

43. Mulquiney PJ, Kuchel PW (1999) Model of 2,3-bisphosphoglycerate metabolism in the human erythrocyte based on detailed enzyme kinetic equations: computer simulation and metabolic control analysis. Biochem J 342 Pt 3: 597-604.

44. Rider CC, Taylor CB (1974) Enolase isoenzymes in rat tissues. Electrophoretic, chromatographic, immunological and kinetic properties. Biochim Biophys Acta 365: 285-300.

45. Wold F, Ballou CE (1957) Studies on the enzyme enolase. I. Equilibrium studies. J Biol Chem 227: 301-312.

46. Garfinkel L, Garfinkel D (1985) Magnesium regulation of the glycolytic pathway and the enzymes involved. Magnesium 4: 60-72.

47. Holzhutter HG, Jacobasch G, Bisdorff A (1985) Mathematical modelling of metabolic pathways affected by an enzyme deficiency. A mathematical model of glycolysis in normal and pyruvate-kinase-deficient red blood cells. Eur J Biochem 149: 101-111.

48. Koster JF, Slee RG, Staal GE, van Berkel TJ (1972) The influence of glucose I,6-diphosphate on the enzymatic activity of pyruvate kinase. Biochim Biophys Acta 258: 763-768.

49. Mc QJ, Utter MF (1959) Equilibrium and kinetic studies of the pyruvic kinase reaction. J Biol Chem 234: 2151-2157.

50. Kahn A, Marie J (1982) Pyruvate kinases from human erythrocytes and liver. Methods Enzymol 90 Pt E: 131-140.

51. Albe KR, Butler MH, Wright BE (1990) Cellular concentrations of enzymes and their substrates. J Theor Biol 143: 163-195.

52. Rozengurt E, Jimenez de Asua L, Carminatti H (1969) Some kinetic properties of liver pyruvate kinase (type L). II. Effect of pH on its allosteric behavior. J Biol Chem 244: 3142-3147.

53. Zewe V, Fromm HJ (1965) Kinetic Studies of Rabbit Muscle Lactate Dehydrogenase. Ii. Mechanism of the Reaction. Biochemistry 4: 782-792.

54. Borgmann U, Moon TW, Laidler KJ (1974) Molecular kinetics of beef heart lactate dehydrogenase. Biochemistry 13: 5152-5158.

55. Wang CS (1977) Inhibition of human erythrocyte lactate dehydrogenase by high concentrations of pyruvate. Evidence for the competitive substrate inhibition. Eur J Biochem 78: 569-574.

56. Uldry M, Thorens B (2004) The SLC2 family of facilitated hexose and polyol transporters. Pflugers Arch 447: 480-489.

57. Thorrez L, Van Deun K, Tranchevent LC, Van Lommel L, Engelen K, et al. (2008) Using ribosomal protein genes as reference: a tale of caution. PLoS One 3: e1854.

58. Gambhir A, Korke R, Lee J, Fu PC, Europa A, et al. (2003) Analysis of cellular metabolism of hybridoma cells at distinct physiological states. J Biosci Bioeng 95: 317-327.
